# Sestrin2 Phosphorylation by ULK1 Induces Autophagic Degradation of Mitochondria Damaged by Copper-Induced Oxidative Stress

**DOI:** 10.3390/ijms21176130

**Published:** 2020-08-25

**Authors:** Heejeong Kim, Byeong Tak Jeon, Isaac M. Kim, Sydney J. Bennett, Carolyn M. Lorch, Martonio Ponte Viana, Jacob F. Myers, Caroline J. Trupp, Zachary T. Whipps, Mondira Kundu, Soonkyu Chung, Xinghui Sun, Oleh Khalimonchuk, Jaekwon Lee, Seung-Hyun Ro

**Affiliations:** 1 Department of Biochemistry, University of Nebraska-Lincoln, Lincoln, NE 68588, USA; hkim7@unl.edu (H.K.); bjeon3@unl.edu (B.T.J.); kimmh0831@gmail.com (I.M.K.); Sydney.Townsend14@huskers.unl.edu (S.J.B.); lorchcar@grinnell.edu (C.M.L.); vianamp@huskers.unl.edu (M.P.V.); jacob.myers@scranton.edu (J.F.M.); caroline.trupp@gmail.com (C.J.T.); zawhipps@hcfalcons.org (Z.T.W.); xsun17@unl.edu (X.S.); okhalimonchuk2@unl.edu (O.K.); jlee7@unl.edu (J.L.); 2 Department of Developmental Neuroscience, Munroe-Meyer Institute, University of Nebraska Medical Center, Omaha, NE 68198, USA; 3 Department of Biology, Grinnell College, Grinnell, IA 50112, USA; 4 Driskill Graduate Program in Life Sciences, Feinberg School of Medicine, Northwestern University, Chicago, IL 60611, USA; 5 Department of Chemistry, The University of Scranton, Scranton, PA 18510, USA; 6 Departments of Pathology and Cell and Molecular Biology, St. Jude Children’s Research Hospital, Memphis, TN 38105, USA; mondira.kundu@stjude.org; 7 Department of Nutrition, University of Massachusetts, Amherst, MA 01003, USA; soonkyuchung@umass.edu

**Keywords:** autophagy, ULK1, Sestrin2, phosphorylation, mitochondria, ATP5A

## Abstract

Selective autolysosomal degradation of damaged mitochondria, also called mitophagy, is an indispensable process for maintaining integrity and homeostasis of mitochondria. One well-established mechanism mediating selective removal of mitochondria under relatively mild mitochondria-depolarizing stress is PINK1-Parkin-mediated or ubiquitin-dependent mitophagy. However, additional mechanisms such as LC3-mediated or ubiquitin-independent mitophagy induction by heavy environmental stress exist and remain poorly understood. The present study unravels a novel role of stress-inducible protein Sestrin2 in degradation of mitochondria damaged by transition metal stress. By utilizing proteomic methods and studies in cell culture and rodent models, we identify autophagy kinase ULK1-mediated phosphorylation sites of Sestrin2 and demonstrate Sestrin2 association with mitochondria adaptor proteins in HEK293 cells. We show that Ser-73 and Ser-254 residues of Sestrin2 are phosphorylated by ULK1, and a pool of Sestrin2 is strongly associated with mitochondrial ATP5A in response to Cu-induced oxidative stress. Subsequently, this interaction promotes association with LC3-coated autolysosomes to induce degradation of mitochondria damaged by Cu-induced ROS. Treatment of cells with antioxidants or a Cu chelator significantly reduces Sestrin2 association with mitochondria. These results highlight the ULK1-Sestrin2 pathway as a novel stress-sensing mechanism that can rapidly induce autophagic degradation of mitochondria under severe heavy metal stress.

## 1. Introduction

Sestrins are stress-responsive proteins that accumulate in cells and tissues in response to genotoxic agents, oxidative stress, and hypoxia [1,2]. In mammals, the Sestrin family is comprised of three closely related members, Sestrin1 (Sesn1 or PA26), Sestrin2 (Sesn2 or Hi95), and Sestrin3 (Sesn3) [3,4]. Sestrin1 is predominantly expressed in cardiac and skeletal muscles, whereas Sestrin2/3 are more commonly expressed in the liver, adipose tissue, and the brain [2,5,6]. *Drosophila melanogaster (Drosophila)* and *Caenorhabditis elegans (C. elegans)* have single Sestrin in their genomes, namely *dSesn* and *cSesn*, respectively. Like mammalian Sestrins, *dSesn* is highly expressed in the cardiac and skeletal muscle and the fat body [4]. Sestrins possess two important functions that can contribute to protecting cells and tissues against environmental stress and aging. First, Sestrins can function as antioxidants, promoting regeneration of peroxiredoxins—one of the major ROS scavengers in cells [7,8,9]. More importantly, Sestrins can stimulate autophagy by inhibiting rapamycin-sensitive mTORC1 signaling complex comprising mTOR and Raptor [10] via AMPK kinase activation or through binding with GTPase activating proteins towards Rags complex (GATOR)2 (MIOS/WDR24/SEH1L/WDR59/SEC13) regulatory complex [11,12]. Autophagy induction also contributes to the suppression of ROS, because it can eliminate dysfunctional mitochondria that produce a pathogenic level of oxygen radicals [13,14]. Additionally, Sestrins induce the expression of antioxidant response-regulating transcription factor Nrf2 through autophagy-mediated degradation of its partner molecule Keap1 [15].

Autophagy, a highly conserved process among plants, eukaryotes, and metazoan, degrades and recycles damaged proteins and organelles and also unwanted cellular aggregates in response to stress conditions [16]. Proper autophagy is important for cellular homeostasis, as its deregulation is associated with diverse metabolic pathologies in both white and brown adipocytes [17,18,19,20]. Mitochondria-enriched metabolic tissue-specific loss of autophagy in mice and humans causes lipid accumulation, unbalanced cytokine secretion, inflammation, and metabolic dysfunction [21,22]. In contrast, autophagy induction by an mTORC1 inhibitor, rapamycin, is able to protect metabolic tissue from the pathogenic levels of ROS produced by dysfunctional mitochondria [23]. Considering that Sestrins are potent inducers of autophagy [24] and that *dSesn*-deficient flies and *Sesn2* knockout (KO) mice are defective in autophagy function [4,15,25], it is plausible that Sestrin2-regulated autophagy is beneficial for metabolic tissue homeostasis and may protect against metabolic dysfunction. *dSesn*-null mutant flies exhibit multiple age-associated degenerative pathologies, including mitochondrial dysfunction, cardiac dysfunction and muscle degeneration. Mitochondrial dysfunction and accompanying fat accumulation underlie these pathologies [4], and are well correlated with the fat body- and muscle-specific expression pattern of *dSesn*. Importantly, the above pathologies can be mitigated by administration of antioxidants and autophagy inducers such as metformin and rapamycin [4]. Other genetic studies also suggest that the ROS- and autophagy-regulating functions of Sestrins are important for the fat body and muscle homeostasis in flies [4,26]. Consistent with the postulated tissue-protective role of Sestrins are the reports that Sestrins maintain cellular homeostasis by inducing autophagy and suppressing ROS in the muscle tissue [27,28]. Sestrin2 associates with autophagy kinase ULK1 and binds to the ubiquitin-binding adaptor protein p62 (also known as SQSTM1) to activate autophagy [29,30]. The autophagy activation is significantly blocked when Sestrin2 KO mouse embryonic fibroblast (MEF) cells are treated with rapamycin [25].

Copper (Cu) is an essential transition metal, micronutrient, as well as a catalytic cofactor required for a variety of cellular functions in metazoans [31]. Facile redox cycling of Cu between two oxidation states, Cu^+^ [Cu (I)] and Cu^2+^ [Cu (II)], can associate with generation of deleterious ROS [32,33,34,35]. When Cu is in excess, oxidative stress caused by Cu-catalyzed ROS is a well-established mechanism of mitochondrial damage [36,37,38], metabolic dysfunction [39,40], and autophagy induction [41,42] in the cells and tissues. Indeed, Cu has been shown to form bioactive complexes with various compounds and signaling molecules—thiosemicarbazone (Dp44mT) [43], HYF127c [44], BNMPH [45], Casiopeina III-ia [46], ETDPA [47], and ULK kinases [42]—to induce autophagy. This feature is now being exploited as an attractive anti-cancer treatment strategy.

Selective autophagic degradation of damaged mitochondria, or mitophagy, was originally identified as a response to mild mitochondria-damaging stress, such as glucose starvation [48,49]. Since then, two major categories of mitophagy induction have been established. In the first category, ubiquitin-dependent degradation of damaged mitochondria is mediated by E3 ligase PINK1- and Parkin-mediated autophagy. When mitochondria is damaged, ubiquitinated mitochondria can associate via PINK1-Parkin pathways with the adaptor protein p62, eventually degrading in autophagosomes [50]. Evidence of a second category, ubiquitin-independent or PINK1-Parkin-independent mitophagy pathways, emerged over the last decade [51,52,53]. Upon specific stress conditions including oxidative stress and hypoxia, or upon treatment of potent mitochondria-damaging agents such as rotenone, staurosporine, and 6-hydroxy dopamine, mitochondria adaptor proteins including Nix [54], Bnip3 [55], Fundc1 [56], Cardiolipin [57], and FKBP8 [58] can directly associate with the autophagosome marker protein microtubule-associated protein 1 light chain 3 (LC3, encoded by MAP1LC3B gene) to induce degradation of damaged mitochondria. Until recently, what has been known is that the outer membrane of severely damaged mitochondria can be ruptured by Parkin-mediated proteasome activity, and the inner mitochondria membrane protein Prohibitin2, only one identified so far, can function as a mitophagy adaptor [59]. However, these reports mainly focused on mitochondrial fusion with autophagosome, and the mechanistic understanding of its upstream effectors remains limited. Regulation of ULK1 kinase by AMPK or mTORC1 has been proposed as one of the upstream signals in mitophagy induction through PINK1-Parkin-dependent pathways [60,61,62]. However, the molecular events preceding the induction of ubiquitin-independent mitophagy and the possible role of Sestrin2 as a rheostat in triggering the degradation of damaged mitochondria through mitophagy induction has not been explored.

In the present study, we have identified that phosphorylation of stress-sensing protein Sestrin2 by ULK1 at Ser-73 and Ser-254 acts as the upstream induction signal for autophagic degradation of mitochondria in response to Cu-induced ROS. Furthermore, we have identified ATP5A subunit of the respiratory complex V as a novel mitochondria adaptor that can directly associate with LC3 upon Cu-ROS stress to promote mitophagy in both ubiquitin-independent and PINK1-Parkin-independent manner. Our findings provide new insights into the molecular mechanism of Sestrin2-induced mitophagy and may aid in the development of Sestrin2-targeting therapies to manipulate mitophagy in human diseases and aging.

## 2. Results

### 2.1. Sestrin2 Interacts with Mitochondrial Protein ATP5A Through Its C-Terminal Domain 

**Figure 1:**
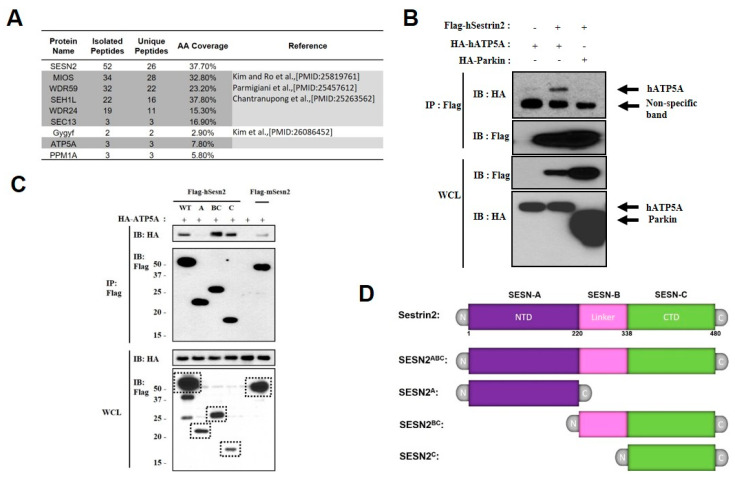
Mitochondrial protein ATP5A interacts with Sestrin2 through C-terminal domain of Sestrin2. (**A**) Mass spectrometry (MS) analysis of Sestrin2-tandem affinity purification (TAP) products identified mitochondrial protein ATP5A (gene name: atp5a1, ATP synthase alpha subunit 1) as proteins that show the strong physical association with Sestrin2. Sestrin2 TAP products from human mammary epithelial cells (MCF-10A) were analyzed by MS-MS. The number of peptide hits for Sestrin2 and previously published binding partners are listed. ATP5A is identified as one of the strong interacting partners (shaded in gray). (**B**) ATP5A is associating with Sestrin2 in mammalian cells. HEK293 cells were transfected with plasmids expressing indicated proteins. FLAG-tagged human ATP5A was immunoprecipitated (IP) from whole cell lysates (WCL) and subjected to immunoblotting (IB) using HA antibody. HA-tagged Parkin from the PINK1-Parkin-mediated mitophagy pathway was used as a non-interacting control with human Sestrin2. (**C**) HEK293 cells were co-transfected with FLAG-tagged wild-type (WT) human Sestrin2 (SESN2^ABC^), truncated human Sestrin2 proteins, FLAG-empty vector, or mouse Sestrin2, and with HA-tagged ATP5A and subjected to immunoprecipitation (IP) using FLAG-conjugated beads. Whole cell lysates and purified immunocomplex were analyzed by immunoblotting (IB). Numbers denote corresponding molecular weights in kDa. (**D**) Diagram depicting the domain structure of WT and truncated mutants of human Sestrin2 used in (**C**).

Our previous proteomic data suggest that human Sestrin2 (hSestrin2) associates with several inner and outer mitochondria membrane proteins, even under basal conditions. To directly validate these findings, we used tandem affinity purification (TAP)-mass spectrometry (MS) analysis. MCF-10A breast epithelial cells were transfected transiently with pBABE-FLAG-SBP-Sestrin2, and then cell lysates were immune-purified with either streptavidin-conjugated or FLAG peptide-conjugated resin and analyzed by liquid chromatography-electrospray ionization-tandem mass spectrometry (LC-ESI-MS/MS) [12]. Among over 150 verified Sestrin2 binding proteins, we were able to identify the four top coverage mitochondrial proteins, such as mitochondrial ATP synthase, F_1_ complex, alpha subunit 1 (ATP5A), sulfide quinone oxidoreductase (SQRDL), Tu translation elongation factor, mitochondrial (TUFM), and acyl-CoA synthetase long-chain family member 5 (ACSL5), residing in inner or outer mitochondria membranes (Figure 1A). Because MS analysis of Sestrin2 binding partners in human breast epithelial cells used whole cell lysates, it is possible to detect arbitrary non-specific association with mitochondria proteins translated from ER-Golgi and trapped in cytosol before translocating into the mitochondria. Therefore, we isolated mitochondria and immune-purified endogenous Sestrin2 using verified Sestrin2-specific antibody and followed with LC-ESI-MS/MS mass spectrometry analysis in HEK293 cells. In these experiments, only ATP5A was in the detectable range among Sestrin2′s candidate interacting partners. Next, we co-expressed FLAG-tagged Sestrin2 with HA-tagged human ATP5A or HA-Parkin in HEK293 cells. We observed a strong association of Sestrin2 with ATP5A but not Parkin (Figure 1B). This suggests that Sestrin2 is unlikely to be directly involved in PINK1-Parkin mitophagy mechanism. We wondered which domain of Sestrin2 would be responsible for ATP5A interaction. To that end, we interrogated the ability of epitope-tagged N-terminal domain (NTD) of Sestrin2 (SESN-A, AA 1-220), loop linker region (Linker) of Sestrin2 (SESN-B, AA 221-338), and C-terminal domain (CTD) of Sestrin2 (SESN-C, AA 339-480) to interact with ATP5A. Only SESN-C domain strongly associated with ATP5A (Figure 1C,D).

### 2.2. Sestrin2 Is Associated with Mitochondria under Condition of the Organelle-Damaging Stress

**Figure 2:**
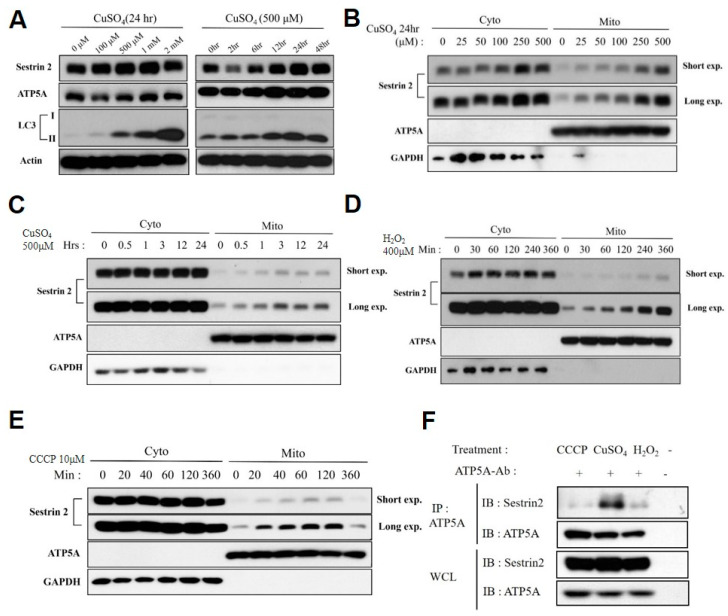
Autolysosome (LC3-II) is accumulated and endogenous Sestrin2 is robustly associated with mitochondria upon chronic treatment with copper. (**A**) Sestrin2, ATP5A, and autophagosome markers LC3-I and II were measured by immunoblotting upon copper (Cu) treatment in indicated concentration and time, as indicated in HEK293 cells. Sestrin2 was induced and associating with mitochondria in (**B**) a dose-dependent manner and (**C**) in a time-dependent manner upon CuSO_4_ treatment. Sestrin2 was induced and associating with mitochondria by (**D**) oxidative stress reagent H_2_O_2_ and by (**E**) mitochondrial membrane un-coupler CCCP treatment in a time-dependent manner. (**F**) HEK293 cells were treated with 10 μm CCCP for 2 h, 500 μm CuSO_4_ for 6 h, or 400 μm H_2_O_2_ for 6 h to maximize endogenous Sestrin2 expression. Then, endogenous ATP5A was immunoprecipitated using protein G/A-conjugated anti-ATP5A antibody. Empty protein G/A beads (−) with no treatment condition were used as negative control. Both anti-ATP5A immunocomplex (IP) and whole cell lysates (WCL) were assayed through immunoblotting (IB).

In previous studies, most mitochondria-signaling proteins, such as Nix, Bnip3, Fundc1, Cardiolipin, and FKBP8, were reported to associate with the outer mitochondrial membrane (OMM) upon carbonyl cyanide m-chlorophenyl hydrazone (CCCP) or H_2_O_2_ treatment, triggering the association with autophagosomes [51,58,63]. On the other hand, in the case of Prohibitin2-mediated mitophagy, this inner mitochondrial membrane (IMM)-resident protein can directly associate with LC3 in autophagosome [59]. In *C. elegans*, Cu-induced ROS robustly induced *cSesn* [64] that is subsequently regulating mitochondrial functions via direct association with mitochondria [65]. Therefore, we tested whether Sestrin2 is associating with mitochondria upon various mitochondria-damaging stresses, including Cu-induced heavy metal stress [66], oxidative stress H_2_O_2_ [62], and mild uncoupling with CCCP [50]. We observed that ROS generated by excessive Cu induced Sestrin2 and, subsequently, autophagy in both concentration- and time-dependent manner (Figure 2A). Among various mitochondrial stress conditions, Cu up to 500 µM sequestered Sestrin2 by associating with mitochondria for up to 24 h (Figure 2B,C). H_2_O_2_ treatment up to 400 µM caused oxidative stress and sequestered Sestrin2 by associating with mitochondria for up to 6 h (Figure 2D), but we could not measure longer time points due to cytotoxicity. CCCP treatment up to 10 µM triggered more acute mitochondrial association of Sestrin2 up to 6 h (Figure 2E) than CuSO_4_ treatment. These data raise the possibility that Sestrin2 may be mediating mitophagy induction by dynamically associating with mitochondria via binding to mitochondria adaptor proteins (Figure 1A)—distinctively under different levels of mitochondrial stress conditions. Next, we assessed the Sestrin2-ATP5A association under the conditions of CCCP, CuSO_4_, and H_2_O_2_ stress. Remarkably, we observed the strongest association between Sestrin2 and ATP5A in the CuSO_4_-treated group (Figure 2F).

### 2.3. Phosphorylated Form of Sestrin2 Is Associated with the OMM of Cu-ROS-Damaged Mitochondria 

**Figure 3:**
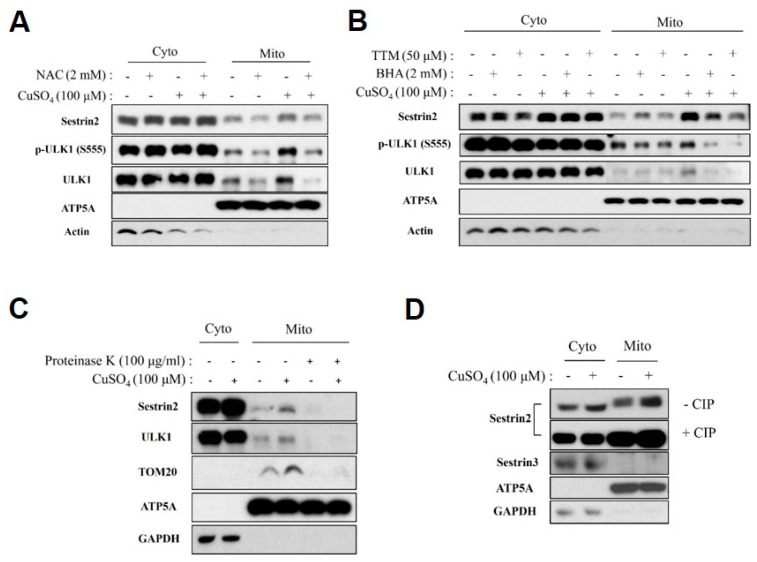
Phosphorylated form of Sestrin2 is associated with the outer mitochondrial membrane upon oxidative damage caused by Cu-induced ROS. (**A**) HEK293 cells were treated with 100 μm CuSO_4_ for 24 h with or without 2 mM antioxidant N-acetyl cysteine (NAC), then the cytosolic and mitochondria fractions were isolated and subjected to immunoblotting with indicated antibodies. (**B**) HEK293 cells were treated with 100 μm CuSO_4_ for 24 h with or without 2 mM butylated hydroxyanisole (BHA) or 50 μM Cu chelator tetrathiomolybdate (TTM), then the cytosolic and mitochondria fraction was isolated and subjected to immunoblotting with indicated antibodies. (**C**) The same fractionation samples from (B) were treated with 100 μg/mL proteinase K for 1 h to perform the mitochondria protein protection assay. 12% SDS-PAGE gel was used to detect the smaller size of proteins in immunoblotting. (**D**) The same fractionation samples from (B) were treated with or without calf intestinal alkaline phosphatase (CIP) for 1 h and assayed through immunoblotting. 8% SDS-PAGE gel was used to ensure enough separation shift for phosphorylated form of Sestrin2.

Cu is a transition metal and its metabolism is known to associate with ROS generation [31,33]. We wondered whether mitochondrial association of Sestrin2 could be ROS-dependent. We isolated cytosolic and mitochondrial fractions from HEK293 cells incubated with or without CuSO_4_ and evaluated mitochondrial association of Sestrin2. Additionally, the cells of interest were treated with antioxidant compounds: 2 mM N-acetyl cystenine (NAC) or 2 mM butylated hydroxyanisole (BHA), or 50 μM Cu chelator tetrathiomolybdate (TTM). Both antioxidants and a Cu chelator were effective in inhibiting mitochondrial association of Sestrin2, indicating that Cu-ROS damage is the likely underlying mechanism of Sestrin2-induced mitophagy in HEK293 cells (Figure 3A,B). Next, we examined the localization of mitochondria-bound Sestrin2. Gradient-purified mitochondria isolated from cells incubated with 100 μM CuSO_4_ or control cells were treated with exogenously added proteinase K and analyzed by immunoblotting. All mitochondria-associated Sestrin2 was degraded by proteinase K, whereas ATP5A remained protected, suggesting that Sestrin2 is mainly associated with the OMM, while ATP5A appears to be protected by the mitochondrial membranes (Figure 3C). Finally, we thought to determine if post-translational modifications such as phosphorylation could be involved in stress-induced association of Sestrin2 with mitochondria. Consistent with previous studies reporting that Sestrin2 is phosphorylated by ULK1 [29], we observed changes in gel mobility of mitochondria-associated Sestrin2, indicative of phosphorylation. Importantly, these changes in electrophoretic mobility were reversed by treatment with calf intestinal alkaline phosphatase (CIP) (Figure 3D). We conclude that the phosphorylated form of Sestrin2 is associated with Cu-ROS-damaged mitochondria in HEK293 cells.

### 2.4. Sestrin2 Is Phosphorylated at Ser-73 and Ser-254 by Autophagy Kinase ULK1 upon Cu-Induced Stress

**Figure 4:**
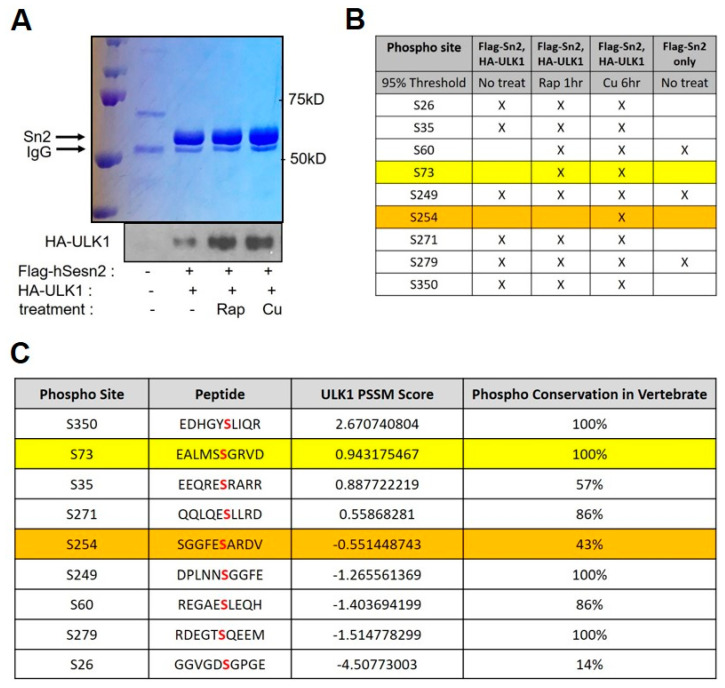
Ser-73 and Ser-254 are Cu-stress-specific phosphorylation sites of human Sestrin2 by ULK1 kinase. (**A**) Coomassie staining of FLAG-tagged human Sestrin2 (hSestrin2) and immunoblotting of HA-tagged ULK1 kinase after being treated with 100 nM rapamycin (Rap) for 1 h or 500 μM CuSO_4_ for 6 h in HEK293 cells. No treatment was used as a control. (**B**) Mass spectrometry analysis of phosphorylated hSestrin2 by ULK1 kinase from coomassie brilliant blue staining samples in (**A**). (**C**) In hSestrin2, ULK1 motif-matching scores and conservation were analyzed by bioinformatics analysis using the motif prediction program. Red letters in Peptide column indicate the specific residue of phosphorylation site (Phospho Site). A detailed algorithm can be found in the Methods Section.

Previous studies reported the existence of phosphorylated form of human Sestrin2 in skeletal muscle after exercise [67]. Moreover, we have also observed previously that Sestrin2 can be phosphorylated by ULK1 [29]. In recent studies on lung carcinoma, ULK kinases were reported to induce autophagy by directly sensing the level of Cu [42]. We therefore interrogated the nature of these modifications and examined their role in the autophagic clearance of damaged mitochondria in our model. We co-expressed FLAG-tagged Sestrin2 with HA-tagged ULK1 in HEK293 cells. To activate ULK1 by mTORC1 inhibition, we treated cells with 100 nM rapamycin (Rap) for 1 h. We also treated cells with 500 µM CuSO_4_ (Cu), for 6 h [66]. We then performed an immunoprecipitation using FLAG affinity resin to enrich Sestrin2 from isolated samples of interest as well as matching controls (Figure 4A). We analyzed posttranslational modifications of Sestrin2 by LC-ESI-MS/MS for phosphorylation and searched for treatment-specific phosphorylations that were not observed in control samples. We identified serine (Ser, S)-73 residue of Sestrin2 (row marked by yellow) as an autophagy-specific phosphorylation site by ULK1 kinase, whereas Ser-254 residue of Sestrin2 (row marked by brown) appears to be a Cu-induced, mitophagy-specific phosphorylation site by ULK1. Ser-73 resides in the N-terminal SESN-A domain and Ser-254 lies within the flexible SESN-B domain (Figure 1D and Figure 4B). We then employed the motif prediction program to analyze ULK1 motif-matching scores (PSSM) and conservation of the residues among Sestrins in vertebrates in question (Figure 4C). Ser-73 is well conserved (100%) but the Ser-254 residue had less conservation (43%) and a lower motif-matching score, suggesting that Ser-73 would be the primary ULK1 phosphorylation target during Cu-ROS-induced mitochondrial stress. Based on our data, it is tempting to speculate that there exists environmental stress-specific phosphorylation of Sestrin2 at Ser-73, and it may function as a rheostat for further downstream signal transduction for autophagy or degradation of mitochondria induction in mammalian cells.

### 2.5. Loss of Phospho-Sestrin2 Inhibits Mitochondria Association with Autophagosome

To confirm that Sestrin2 loss can indeed affect the clearance of mitochondria damaged by Cu-ROS stress, we assessed the ATP5A–LC3 association in Sesn2 (Sn2) WT and KO mouse embryonic fibroblast (MEF) cells after treating them with 500 µM Cu for 6 h. The observed decreased colocalization of mitochondria and autophagosome in the Sestrin2-deleted cells by both the immunofluorescence and immunoblotting (Figure S1A,B) reflects the inhibition of mitochondria–autophagosome association. We further wondered if mutating the Ser-73 and Ser-254 phosphorylation sites to alanine (Ala, A) would affect Sestrin2′s association with Cu-damaged mitochondria. To that end, we have analyzed both cytosolic and mitochondrial fractions from Sestrin2-knockdowned HEK293 cells after transfecting them with RNAi-resistant WT, S73A, or S254A variants of FLAG-Sestrin2 and assessing their association with mitochondria after treatment with 500 µM CuSO_4_ for 0, 6 and 24 h. The mitochondrial association of FLAG-Sestrin2 WT was increased by 42% and 48% after 6 and 24 h of treatment respectively, when compared to the untreated control. By contrast, mitochondrial association of the S73A variant was decreased by 10% and 11% after 6 and 24 h of treatment, respectively. Similarly, mitochondrial association of S254A was decreased by 10% after 6 h post-treatment but showed no appreciable difference after 24 h of Cu treatment (Figure S2A,B). We conclude that phosphorylations of Sestrin2 at Ser-73 and Ser-254 serve as signals to facilitate its mitochondrial association upon Cu-ROS stress.

### 2.6. Sestrin2 Colocalizes with ATP5A in Mitochondria upon Cu-Induced Stress in HEK293 Cells 

**Figure 5:**
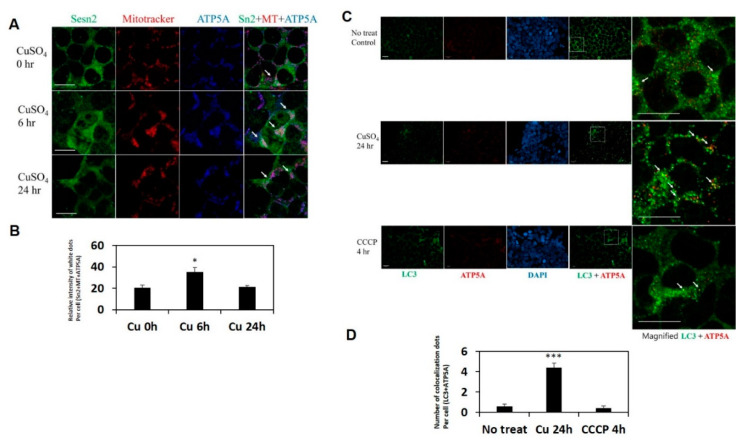
Sestrin2 is strongly colocalized with mitochondria and autophagosomes following Cu toxicity stress. (**A**) HEK293 cells were treated with 500 μm CuSO_4_ for the indicated time in the presence of 100 nM bafilomycin A1 and stained with mitotracker (red) for mitochondria and immunostained with anti-Sestrin2 (green) and anti-ATP5A (blue) antibodies. (**B**) Relative intensity of white dots indicating colocalization between Sestrin2 (Sesn2 or Sn2), mitochondria (MT), and ATP5A was counted by the ImageJ program and quantified as presented in a graph (*n* = 5). (**C**) HEK293 cells were treated with 500 μM CuSO_4_ for 24 h or 50 μm CCCP for 4 h in the presence of pepstatin A and E-64 (10 μg/mL each) and immunostained with anti-LC3 (green), anti-ATP5A (red) antibodies, and DAPI (DNA, blue). (**D**) Number of yellow dots indicating colocalization between LC3 and ATP5A was counted and quantified as presented in a graph (*n* = 5). White arrows indicate the colocalization between indicated fluorescent markers. Data are shown as mean ± s.e.m. * *p* < 0.05, *** *p* < 0.001. *p*-value is from one-way analysis of variance (ANOVA). Scale bars = 200 μm (white).

Apart from Sestrin2 phosphorylation by ULK1, we also determined that Sestrin2 is colocalizing with mitochondria through mitochondria adaptor proteins upon Cu-induced mitochondrial stress. The current view in the field is that autophagy proteins such as Nix and Bnip3 or mitochondria adaptor proteins such as Fundc1, Cardiolipin and FKBP8 directly associate with damaged mitochondria and induce autophagic degradation [51,58,63]. Since we observed strong Sestrin2 induction and mitochondrial association up to 24 h upon treatment with 500 µM CuSO_4_ (Figure 2B,C), we sought to use that same exact condition to assess Sestrin2 association with mitochondria. Our results suggest that Sestrin2 can directly associate with mitochondria in response to Cu stress through adaptor proteins, and ATP5A may be such a protein (Figure 5A,B). When we induced mitophagy by treating cells with 500 µM CuSO_4_, we observed strong colocalization of Sestrin2 and ATP5A at 6 h post-treatment, and gradually declining over 24 h after treatment. We were unable to test mitochondrial association of Sestrin2 in ATP5A-depleted cells, due to the essential role of ATP5A in cellular ATP production [68].

### 2.7. Mitochondria Adaptor Protein ATP5A Contains Several Putative LIR Motifs and Directly Interacts with Autophagosome Marker LC3 

How can two proteins that reside in two different mitochondrial sub-compartments interact? It has been previously reported that LC3 interacting proteins such as p62, ATG32, NBR, and Fundc1 contain the LIR motif, [W/F/Y]XX[L/I/V], which is required for their interaction with LC3 [69]. We speculated that ATP5A might also harbor LIR motifs as a means of interacting with LC3. Upon examination of ATP5A protein sequence, we found five candidate LIR motifs within the translated ATP5A ORF, with at least one motif being perfectly in line with that of the other known LC3-interacting proteins (Figure S3A). We then co-expressed HA- or FLAG-tagged ATP5A with GFP-LC3 and performed IPs using HA- or FLAG-resin. HA- or FLAG-tagged ATP5A robustly associated with GFP-LC3 (Figure S3B,C). By contrast, IP of Sestrin2, which does not have any LIR motifs, did not result in LC3 co-precipitation (Figure S3B,C). These data suggest that Sestrin2 likely associates with LC3 through the mitochondria adaptor protein ATP5A to induce mitophagy.

### 2.8. ATP5A Colocalizes with LC3 upon Cu-Induced Stress in HEK293 Cells 

Next, we posited that endogenous ATP5A could bind to LC3 when phospho-Sestrin2 associates with damaged mitochondria as an induction signal (Figure S3). In line with this notion, we observed a robust colocalization of the endogenous ATP5A and the autophagosome protein LC3 in HEK293 cells challenged with CuSO_4_, but not with the uncoupler CCCP (Figure 5C,D). Although we have observed induction of Sestrin2 and some association of the protein with mitochondria upon treatment with 10 µM CCCP (Figure 2E), we had to use a 5-fold higher concentration of CCCP (50 µM) to ensure robust recruitment of Sestrin2 to mitochondria. We only observed strong colocalization of ATP5A and LC3 in CuSO_4_ treatment up to 24 h. These data suggest that the endogenous phospho-Sestrin2 can associate with mitochondria in both CuSO_4_- and CCCP-treated cells, however the ATP5A–LC3 association is more robust under the condition of Cu stress. In line with this notion, a very recent study reported that Sestrin2 can associate with mitochondria in CCCP-treated cells to promote mitophagy [62] and our data firstly reported a Sestrin2 association with mitochondria to promote mitophagy in copper-treated cells.

### 2.9. Oral Injection of Cu Increases Autophagic Activity in Mouse Liver 

**Figure 6:**
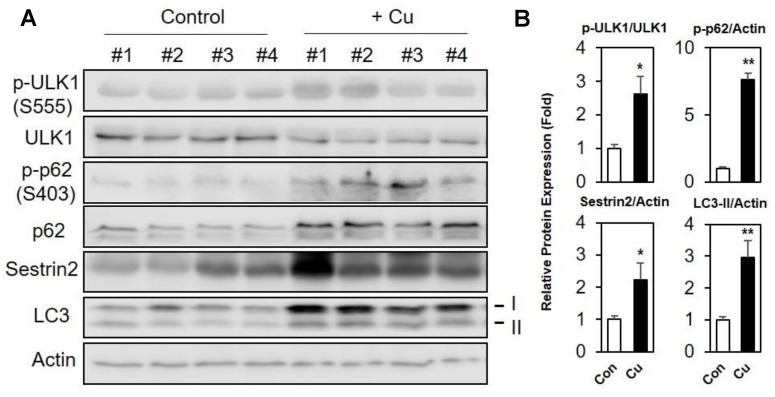
Cu treatment increases expression of Sestrin2 and facilitates ULK1 and p62 phosphorylations in mouse liver. (**A**) C57BL/6 mice (B6 male, 8 weeks old) were injected orally with 0.29 mg/kg CuCl_2_ or saline for 6 h (*n* = 4 for each group). Liver tissues were collected, and total liver lysates were subjected to immunoblot analysis for phospho-ULK1 S555, total ULK1, phospho-p62 S403, total p62, Sestrin2 and LC3 in liver tissue. β-actin (Actin) were used as loading controls. (**B**) Each immunoblot band intensity was analyzed by Image J, as indicated. * *p* < 0.05, ** *p* < 0.01. *p*-value is from Student’s *t*-test.

We sought to test whether autophagy activity is changed in vivo. To that end, we have orally administered a physiological dose of CuCl_2_ (0.29 mg/kg for 6 h) to 8-week-old B6 male mice (n = 4) and analyzed the autophagy markers in the liver by immunoblotting (Figure 6A). The protein expression levels of Sestrin2 were increased 2.2-fold in the liver of Cu-treated mice relative to the control group. Moreover, phosphorylation of ULK1 at Ser-555, a mitophagy-inducing phosphorylation by AMPK, and phosphorylation of ULK1 target protein p62 (Ser-403) were significantly increased (2.6-fold and 7.6-fold, respectively), indicating that ULK1 activity is elevated upon Cu-ROS stress. The 3-fold increase in LC3-II signal in liver tissue of Cu-treated mice also reflects a significant increase in autophagy flux (Figure 6B). These data suggest that the ULK1-Sestrin2 pathway is conserved and induces autophagy in response to copper-induced stress in liver tissues of mice. 

## 3. Discussion

In the present study, we identified a novel selective autophagy induction mechanism for recycling of mitochondria damaged by copper-induced oxidative stress. The mechanism comprises phosphorylation of Sestrin2 by autophagy kinase ULK1, followed by the association of modified Sestrin2 with the damaged mitochondria. Sestrin2 has been previously shown to be a master regulator of autophagy induction through its role in activating AMPK and inhibiting mTORC1 [4,15,24]. Our work describes a new, previously unanticipated role of Sestrin2 in mitophagy induction and provides a potential explanation as to how ULK1 kinase integrates with AMPK and mTORC1 pathways as a feedback mechanism via direct phosphorylation of Sestrin2 and thus orchestrates the autophagic degradation of mitochondria dependent on the level of its damage [70]. Sestrin2 is known to mediate the clearance of dysfunctional mitochondria through association with ULK1 and p62 [29,71,72]. When Sestrin2 is deficient, cells and tissues are unable to adapt to mitochondria-damaging stress and accumulate excessive amounts of dysfunctional mitochondria [4]. Our data indicate that by controlling phosphorylation of Sestrin2 at Ser-73 and Ser-254, ULK1 induces autophagic degradation of mitochondria, at least in part, when cells are challenged by Cu-induced oxidative stress. The ULK1–Sestrin2 axis-mediated direct activation of mitophagy can be further enhanced upon indirect upstream modulation of ULK1 at Ser-555 (activating modification) and Ser-757 (inhibitory modification) via AMPK and mTORC1 respectively, to further facilitate mitochondrial degradation [60,61,73]. 

Although ULK1 is the only known autophagy kinase among 16 essential autophagy genes in mammalian cells [72,74,75], identification of its downstream partners has drawn a great deal of attention and efforts in the autophagy research community due to its quintessential role in autophagy initiation [76]. These efforts led to the identification of a repertoire of ULK1 targets including several autophagy adaptors, regulators, and effectors, such as AMBRA1 [77], Beclin-1 [78], ZIPK [79], Atg9 [80], and p62 [29,30]. In addition, previous studies established that ULK1 can phosphorylate Sestrin2 and phosphorylated Sestrin2 activates ULK1 activity toward p62 phosphorylation as an autophagy induction signal [29]. Consistent with our work, Cu has been suggested to modulate autophagy signaling through direct interaction with ULK kinases in lung adenocarcinoma [42]. Moreover, Sestrins were reported to colocalize with mitochondria and regulate mitochondrial functions in *C. elegans* and human lung adenocarcinoma A549 cells [65]. 

We showed that phosphorylation of Sestrin2 at Ser-73 and Ser-254 by ULK1 serves as an important selective autophagy induction signaling mechanism when mitochondria are damaged by Cu-induced oxidative stress. Wu and others have reported that ULK1 can be translocated into the mitochondria and induce mitophagy by associating with mitochondria adaptor protein Fundc1 [81]. In our previous report, we have established that Sestrin2 can be phosphorylated by ULK1 and act as a strong activator of ULK1 through direct binding [29]. Together with our new findings, these data suggest that phosphorylated Sestrin2 could act a mediator, transmitting mitophagy induction signal from ULK1 to mitochondria and autophagosome. We identified ATP5A as a likely signaling adaptor protein for mitophagy. Such a moonlighting role for this core component of mitochondrial ATP synthase that resides in the IMM/matrix sub-compartment of mitochondria is not necessarily unprecedented. Indeed, a previous study reported that an IMM protein, Prohibitin2, can be exposed when mitochondria are severely damaged by an oligomycin/antimycin A cocktail and mediates Parkin-mediated mitophagy initiation [59]. Furthermore, another study reported that Sestrin2 can activate ULK1-mediated phosphorylation of Beclin1 at Ser-14 to induce Parkin-mediated mitophagy [62]. However, how can matrix-residing ATP5A be associated with the OMM-localized Sestrin2? Our current model considers the following scenarios. First, Cu-induced oxidative stress may be attenuating mitochondrial protein import, thereby causing newly synthesized ATP5A to sequester in the outer membrane of mitochondria *en route* to its final destination. As mitochondrial proteins are imported in an unfolded state [82], such translocation arrest may result in the prolonged exposure of ATP5A’s LIR motif and recruitment of factors like Sestrin2 and LC3 for subsequent mitophagy initiation. A similar chain of events has been reported for several mitochondria-targeted proteins, including PINK1 in mammalian cells and ATFS-1 in *C. elegans* [83]. A less likely scenario for ATP5A association with mitophagy machinery components would be massive membrane permeability/damage, resulting in exposure of mitophagy adaptors like ATP5A, and their recognition by relevant mitophagy-priming factors. Furthermore, previous studies did not provide information on the upstream induction signal of ULK1 and merely assumed that AMPK and mTORC1 would be upstream of the components in question. Based on the results of our study, we speculate that Sestrin2 could be a conduit of autophagy induction, sensing both AMPK/mTORC1 and downstream ULK1 pathways in selective induction of mitophagy.

Our work demonstrates the importance of Sestrin2 phosphorylation and association with mitochondria in vitro and in vivo as a rheostat for mitophagy induction during mitochondria-damaging stress (Figure S4). We have identified novel and unique phosphorylation sites of Sestrin2 by ULK1 specific to different levels of mitochondria-damaging agents, as well as its association with mitochondria in both concentration- and time-dependent manner (Figure S4). These studies warrant further examination of (i) Sestrin2 induction and its phosphorylation by ULK1, (ii) mechanistic aspects of Sestrin2′s mitochondrial association via ATP5A upon mitochondrial damage, and (iii) physiological significance of our findings in normal and pathological states.

## 4. Materials and Methods 

### 4.1. Plasmids, Antibodies, and Chemicals 

FLAG-Sesn2^WT^ (SESN2^ABC^), FLAG-Sesn2^∆BC^(SESN2^A^), FLAG-Sesn2^∆A^(SESN2^BC^), and FLAG-Sesn2^∆AB^ (SESN2^C^) were subcloned into pcDNA3.1 plasmid (Invitrogen, Carlsbad, CA, USA) [29]. HA-ULK1 WT, HA-ULK1 KI (kinase-inhibited), HA-Parkin, and GFP-LC3 were from Dr. K.L. Guan (UC-San Diego, San-Diego, CA, USA), Dr. M. Aghajan (Ionis Pharm. Inc., Carlsbad, CA, USA), and Addgene [73]. Myc-ULK1 WT and Myc-ULK1 KD (kinase dead) were a generous gift from Dr. D. H. Kim (University of Minnesota, Minneapolis, MN, USA). HA-hSestrin2 and FLAG-hSestrin2 were a generous gift from Dr. J. H. Lee (University of Michigan, Ann Arbor, MI, USA). FLAG-hSestrin2 S73A and S254A mutants were produced using a Phusion Site-Directed Mutagenesis Kit (F541) and confirmed by DNA-sequencing using primers from Eurofins Genomics (Lousville, KY, USA). pCMV3-HA-hATP5A (HG14419-NY) and pCMV3-FLAG-mATP5A (MG59917-CF) were purchased from Sino Biological (Wayne, PA, USA). Antibodies to detect ULK1 (4773), p-ULK1 S555 (5869), p62/SQSTM1 (5114), p-p62/SQSTM1 S403 (39786), and LC3 (3868) were from Cell Signaling Technology. Anti-Sestrin2 (10795-1-AP) antibody was from Proteintech (Rosemont, IL, USA). Anti-ATP5A was from Abcam (Cambridge, MA, USA). Anti-hemagglutinin (HA) antibody (3F10) was from Roche (Basel, Switzerland). Anti-Actin, Anti-GAPDH, and anti-Tubulin antibodies were from Developmental Studies Hybridoma Bank (Iowa City, IA, USA). Anti-FLAG (M2) antibody was from Sigma (Sigma-Aldrich, St. Louis, MO, USA). Mitotracker Red was purchased from Invitrogen. TTM (ammonium tetrathiomolybdate, (NH_4_)_2_MoS_4_, 323446), BHA (B1253), and NAC (A7250) were purchased from Sigma. CuSO_4_ (C1297), CuCl_2_ (451665), H_2_O_2_ (H1009), and CCCP (C2759) were purchased from Sigma.

### 4.2. Cell Culture and DNA Transfection 

HEK293 and MEF cells were cultured in Dulbecco’s modified Eagle’s medium (DMEM, Invitrogen) containing 10% fetal bovine serum (FBS) and penicillin/streptomycin at 37 °C in 5% CO_2_. *Sesn2**^+/+^*** (WT) and *Sesn2^−/−^* (KO) MEF cells were previously described [25]. For transient expression of proteins, HEK293 cells were transfected with purified plasmid constructs using Lipofectamin 2000 (Invitrogen) according to the manufacturer’s protocol. Cells were harvested two days after transfection for immunoblotting, immunoprecipitation, immunocytochemistry, or other biochemical assays.

### 4.3. Immunocytochemistry 

Two days after transfection, HEK293 cells were seeded onto glass coverslips. On the following day, HEK293 or MEF cells were washed with PBS and fixed with 4% paraformaldehyde. After permeabilizing the cells with 0.3% Triton X-100, they were incubated overnight with primary antibodies and mitotracker red. The cells were then washed with PBS and were incubated with Alexa Fluor-conjugated secondary antibodies (Invitrogen) for 30 min and counterstained with DAPI (Invitrogen) [84]. Images were taken with a Leica DMI4000B fluorescence microscope (Leica Microsystems, Wetzlar, Germany). The fluorescence intensity was measured with a Synergy H1 Hybrid Multi-Mode Reader (BioTek Instruments, Winoosk, VT, USA). Relative fluorescence intensity is the fold of the mean fluorescence intensity of the controls.

### 4.4. Immunoprecipitation 

Cell lysates were prepared in a lysis buffer (20 mM Tris-Cl pH 7.5, 150 mM NaCl, 1 mM EDTA, 1 mM EGTA, 2.5 mM NaPPi, 1 mM β-glycerophosphate, and 1 mM Na_3_VO_4_) containing 0.3% CHAPS (Sigma, C3023) and protease inhibitor cocktail (Roche, 11873580001), and immunoprecipitated with anti-HA (Sigma, A2095) or anti-FLAG (Sigma, A2220) agarose bead or other antibodies conjugated to a protein G/A bead (Calbiochem, Burlington, MA, USA). The immunocomplexes were then washed four times with the lysis buffer and analyzed through immunoblotting [85].

### 4.5. Calf Intestinal Alkaline Phosphatase (CIP) Assay 

Cell lysates were prepared in the lysis buffer described above and incubated with 1 unit/μL CIP (New England Biolabs, M0290S) at 30 °C overnight [85].

### 4.6. Immunoblotting 

Cell lysates prepared in cell lysis buffer (20 mM Tris-Cl pH 7.5, 150 mM NaCl, 1 mM EDTA, 1 mM EGTA, 2.5 mM sodium pyrophosphate, 1 mM β-glycerophosphate, 1 mM Na_3_VO_4_, 1% Triton-X-100) containing protease inhibitor cocktail (Roche) and phosphatase inhibitor (Sigma), immunocomplexes, and enzymatic reaction mixtures were boiled in SDS sample buffer for 5 min, separated by SDS-PAGE, transferred to PVDF membranes, and probed with primary antibodies. After incubation with secondary antibodies conjugated with HRP, chemiluminescence was detected using an Odyssey Clx imaging system (LI-COR Biosciences, Lincoln, NE, USA) [29]. Raw uncropped images with molecular size marker were displayed in the supplementary information section (Figure S5).

### 4.7. Mitochondria Isolation and Proteinase K Protection Assay 

Mitochondria fractionations were carried out as previously described [81,86]. Briefly, HEK293 cells were collected and resuspended in hypotonic buffer (10 mM KCl, 210 mM sucrose, 70 mM mannitol, 1 mM EDTA, 1 mM EGTA, 1.5 mM MgCl_2_, 10 mM HEPES (pH adjusted to 7.4 with KOH)) containing 2 mM PMSF and protease inhibitor cocktail. After gentle homogenization with a Dounce homogenizer (about 50 times), cell extracts were centrifuged at 3000 *g* for 10 min at 4 °C. The supernatant was removed to an Eppendorf tube and 100 μL was set aside in another Eppendorf tube as the post-nuclear supernatant (PNS). The remaining supernatant was centrifuged at 10,000 *g* for 10 min and the new supernatant was removed and set aside as the cytoplasmic fraction (Cyto). The pellet was washed twice with hypotonic buffer, resuspended in 0.5 mL hypotonic buffer, then layered on top of a step Percoll (GE Healthcare Life Science, Chicago, IL, USA) gradient (2 mL 80%, 4.5 mL 52%, 4.5 mL 26% in a SW40 tube), which was centrifuged at 20,000 *g* for 2 h. A 0.4 mL fraction containing pure mitochondria (Mito) was collected from the interface between the 26% and 52% layers and diluted with 0.8 mL hypotonic buffer. The Mito fraction was centrifuged in an Eppendorf tube at 20,000 *g* for 10 min and washed 3 times with hypotonic buffer. Samples were mixed with 1× loading buffer and heated for 10 min at 100 °C before analyzing by immunoblotting. For the proteinase K protection assay, an aliquot of Cyto or Mito fractions were incubated with 1% (*w*/*v*) Triton X-100 in sucrose/EGTA/MOPS on ice for 3 min. Two hundred micrograms of protein equivalent of Triton X-100-treated or untreated samples were incubated for 15 min at 37 °C with 100 µg/mL (final concentration) proteinase K (Sigma, P2308), dissolved in sucrose/EGTA/MOPS (final volume of 100 µL). The reaction was stopped by adding 10 µL of a 200 mm phenylmethanesulfonyl fluoride solution (in propanol), after which the sample was placed on ice for 20 min. The samples were boiled for 3 min with adding 1× sample buffer, after which another 10 µL of phenylmethanesulfonyl fluoride solution was added, then analyzed by immunoblotting. 

### 4.8. Tandem Affinity Purification-Mass Spectrometry Analysis of Sestrin2 Binding Proteins and Phosphorylation Sites 

Tandem affinity purification (TAP)-MS analysis was performed according to the method that we have formerly described [12]. In brief, MCF10A cells were stably infected with pBABE-FLAG-SBP-Sestrin2 retroviruses. Cells were then cultured to 80% confluency and lysed with buffer containing 0.3% CHAPS. Lysate was first incubated with FLAG antibody-conjugated resin and FLAG-SBP-Sestrin2 was eluted with elution buffer containing 200 ng/μL 3× FLAG peptide. The eluate was further incubated with streptavidin-conjugated resin and FLAG-SBP-Sestrin2 was again eluted with buffer containing 4 mM biotin. The TAP-purified FLAG-SBP-Sestrin2 and associated proteins were trypsin digested and analyzed by LC/ESI MS/MS. Proteins identified in both vector control and FLAG-SBP-Sestrin2 experiments were determined to be false-positive. Phosphorylation sites of human Sestrin2 by ULK1 kinase was analyzed by TAP-MS as previously reported [87,88]. FLAG-tagged human Sestrin2 was co-expressed with HA-tagged ULK1 WT or KI in HEK293T cells for 2 days. Cells were lysed in a buffer containing 40 mM HEPES (Sigma, H7006), pH 7.4, 120 mM NaCl, 1 mM EDTA, 50 mM NaF, 1.5 mM Na_3_VO_4_, 10 mM β-glycerophosphate, and 0.3% CHAPS (Sigma, C3023), supplemented with protease inhibitors (Roche, 11873580001), and FLAG-hSestrin2 was isolated by immunoprecipitation using anti-FLAG antibody. The immunoprecipitated hSestrin2 was resolved by SDS-PAGE, and the hSestrin2 band was subjected to overnight digestion using trypsin (Promega, Madison, WI, USA). Tryptic peptides were extracted from the gel and dried down using a speed vacuum, then resuspended in solvent (97.4:2.5:0.1, water:acetonitrile:formic acid) and analyzed using a Q-Exactive HF mass spectrometer (Thermo Fisher Scientific, Waltham, MA, USA). Tandem MS data for the phosphorylated hSestrin2 peptide was acquired using the HCD mode. Peptide spectra were analyzed by MS/MS using Mascot (Matrix Science, London, UK; Version 2.6.1). We validated MS/MS-based peptide and protein identification using Scaffold (Proteome Software Inc., Portland, OR, USA). Peptide identifications were accepted if the probability was greater than 99% by the Peptide Prophet algorithm.

### 4.9. Matching Scores and Conservation Analysis of ULK1 Motif in Sestrin2 

The ULK1 kinase binding motif was defined by the ULK1 position-specific scoring matrix1 from the Reuben Shaw group’s peptide array data [89]. The amino acid binding probability was normalized by the human proteome amino acid content and then log 2 was applied on the ratio. To ensure the score represent positions with high information content, positions −3, 0, 1, and 2 were used in the scoring. Each Ser/Thr amino acid on Sesn2 was considered as a putative phosphorylation site. For each site, the region surrounding the putative phosphorylation site was extracted and scored using the ULK1 PSSM. Site: the position of the Ser/Thr on SESN2, Peptide: amino acids surround the phosphorylation site (−5 to 4 position), and Score: the score generated based on the ULK1 PSSM.

### 4.10. Oral Administration of Copper in Mice and Liver Tissue Collection 

Copper chloride (CuCl_2_-2H_2_O) stock solution at 38.33 mg/mL was prepared by dissolving in distilled fresh regular water right before use. Cu was orally administered to mice (C57BL/6, male, 8 weeks old) at dose of 0.29 mg/kg for 6 h after diluting the Cu stock solution with distilled water (*n* = 4 per group). Total liver tissue lysates were prepared in RIPA buffer (50 mM Tris-Cl pH 7.4, 150 mM NaCl, 1% sodium deoxycholate, 1% NP-40; 0.1% SDS) containing protease inhibitor cocktail (Roche) and phosphatase inhibitor (Sigma), and protein concentrations were determined by the BCA assay kit (Thermo Fisher Scientific, Waltham, MA, USA). Immunoblot band density was measured by the ImageJ program (National Institute of Health, Bethesda, MD, USA) and normalized by the intensity of the loading control, as indicated.

### 4.11. Statistical Analysis 

Data are presented as mean ± s.e.m. The data were statistically evaluated using Student’s t-test or one-way analysis of variance (ANOVA) with SPSS 25 (IBM Corporation, Armonk, NY, USA). Statistical significance was indicated when *p* < 0.05.

## Supplementary Materials

Supplementary Information can be found at https://www.mdpi.com/1422-0067/21/17/6130/s1.

## Abbreviations

AMPK	AMP-activated protein kinase
mTORC1	mechanistic target of rapamycin complex 1
ULK1	Unc-51-like protein kinase 1
ATP5A	ATP synthase alpha subunit 1, mitochondrial
LC3	microtubule-associated protein 1 light chain 3
ROS	reactive oxygen species
CIP	calf intestinal alkaline phosphatase
MEF	mouse embryonic fibroblasts
HEK293	human embryonic kidney cells 293
