# Supplementary material for: Sestrin2 Phosphorylation by ULK1 Induces Autophagic Degradation of Mitochondria Damaged by Copper-Induced Oxidative Stress
Source: Int J Mol Sci. 2020 Aug 25;21(17):6130. doi: 10.3390/ijms21176130 (PMC7504119; doi:10.3390/ijms21176130)
Supplement: Supplementary file 1 [file ijms-21-06130-s001.pdf]

Supplementary Information

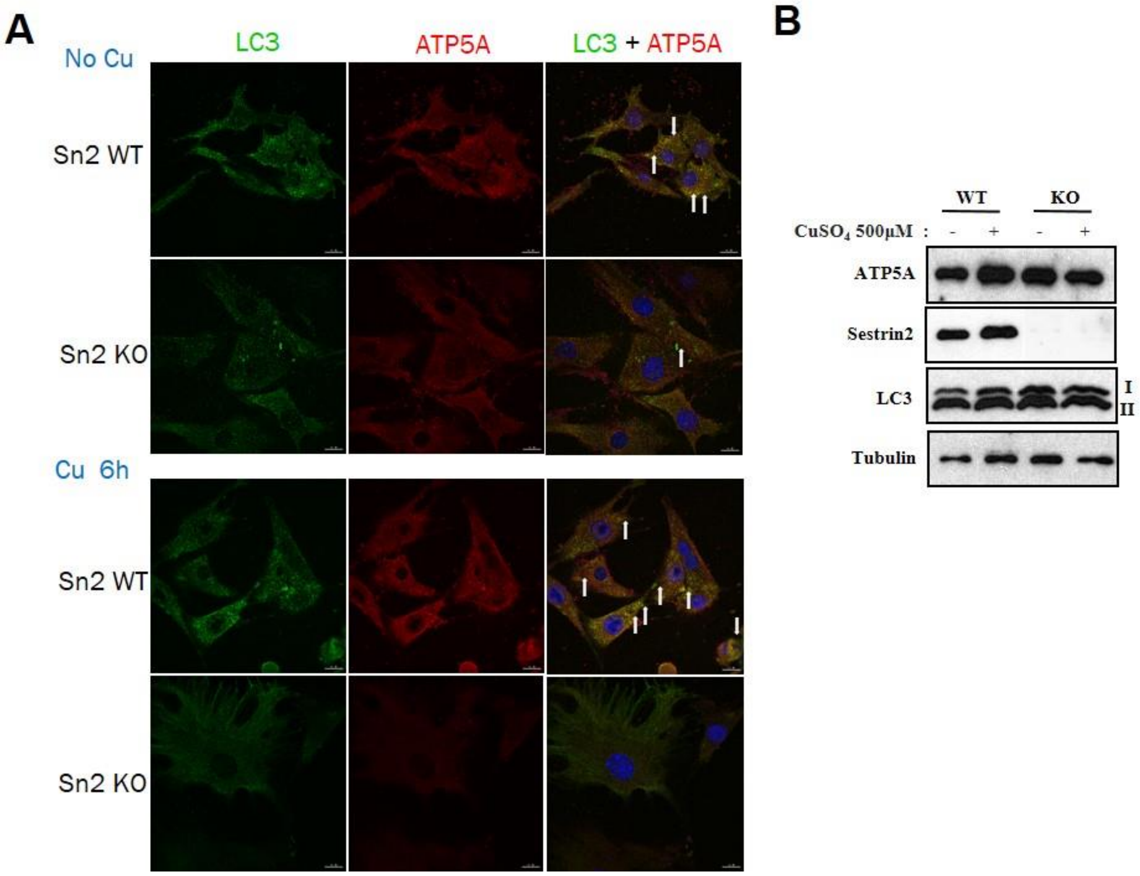

Figure S1. Loss of Sestrin2 inhibits mitochondria-autophagosome association upon Cu-ROS stress in MEF cells. (A) Images of Sesn2 (Sn2) WT and Sn2 KO MEF cells treated with 500 μM $CuSO_4$ for 0 or 6 h. Blue = DAPI for nuclei, red = ATP5A, and green = LC3. Images were collected with a Nikon inverted laser scanning confocal microscope (60X oil objective). Scale bar = 20 μm. Arrows point to spots of colocalization between LC3 and ATP5A, which is indicative of association between autophagosome and mitochondria. **(B)** Immunoblotting of Sn2 WT and KO cells from (A).

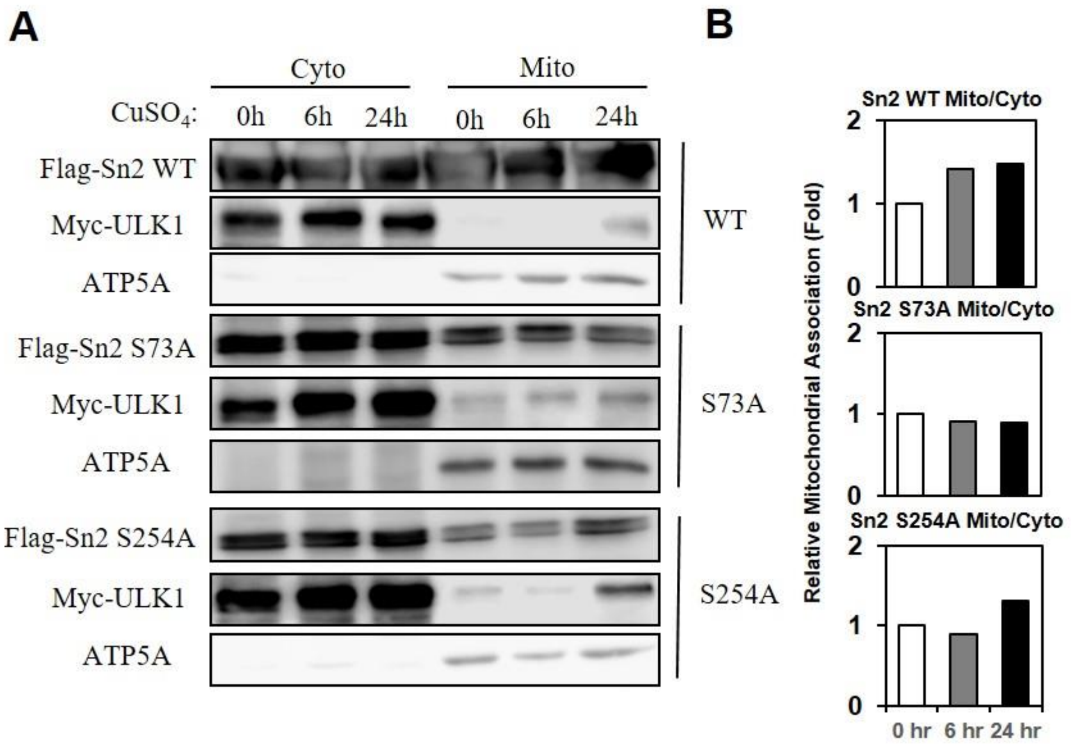

Figure S2. Loss of Sestrin2 phosphorylation at Ser-73 and Ser-254 attenuates its mitochondrial association upon Cu-ROS stress in HEK293 cells. (A) Endogenous Sesn2 (Sn2) was knockdowned in HEK293 cells using human Sestrin2 specific- lentivirus (Sh-hSn2) as previously described. Sestrin2-knockdowned HEK 293 cells were transfected with the Sn2 WT, S73A or S254A mutants and subjected mitochondrial fractionation (Mito) from cytosolic fraction (Cyto) after treated with 500 μM $CuSO_4$ for 0, 6 and 24 h. Fractionated proteins were analyzed by immunoblotting with indicated antibodies. **(B)** Bar graphs showing the relative mitochondrial association of hSn2 WT, S73A and S254A mutants.

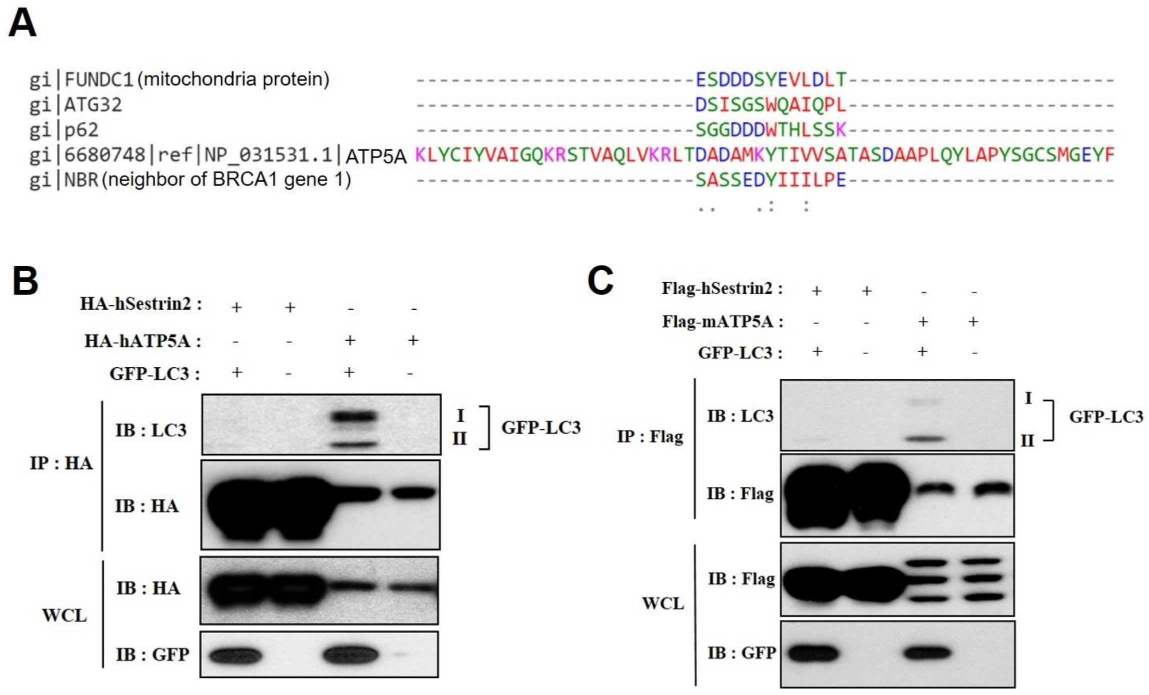

Figure S3. ATP5A but not Sestrin2 physically interacts with LC3 in HEK293 cells. (A) LC3- interacting region (LIR) motif (YXXL) was analyzed in ATP5A and aligned with previously known LIR motif from other LC3 binding proteins using Cluster Omega program. **(B, C)** HEK293 cells were co-transfected with either HA- or FLAG-tagged human Sestrin2, HA-tagged human or FLAG-tagged mouse ATP5A and GFP-LC3, and immunoprecipitation (IP) was performed using HA- or FLAG-conjugated beads. The indicated protein was detected using immunoblotting (IB).

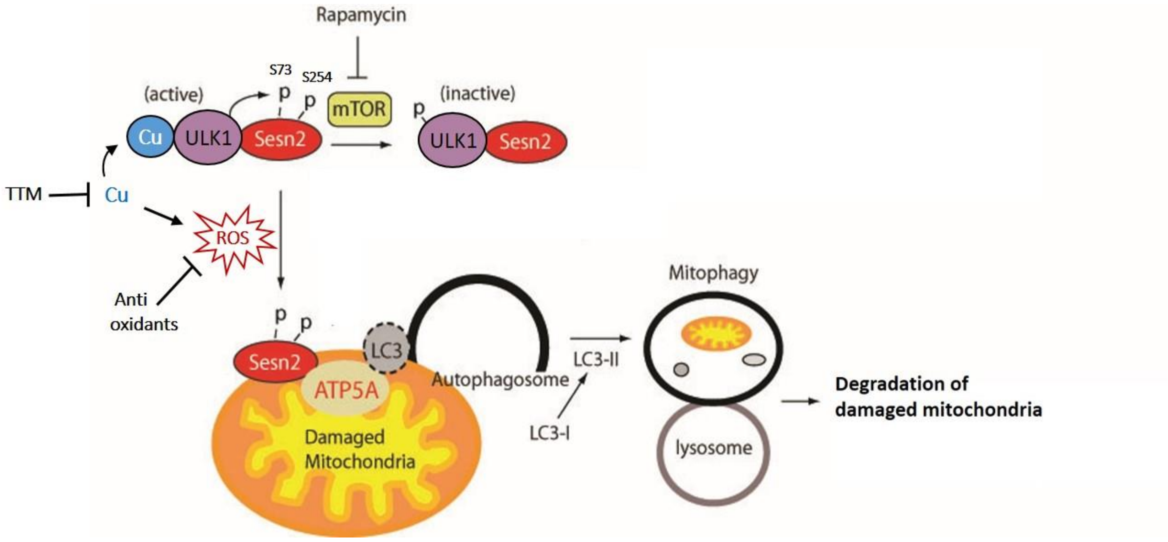

Figure S4. Schematic model of Sestrin2- and ATP5A-mediated mitophagy induced by transition metal stress. Chronic treatment of heavy transition metal Cu generates ROS which activates ULK1-Sestrin2 mitophagy induction signal. Sestrin2 is phosphorylated at Ser-73 and Ser-254 by active ULK1 and then associating with damaged mitochondria. Sestrin2 and mitochondria (ATP5A) association triggers LC3 binding to initiate mitophagy upon Cu-ROS stress. Degradation of damaged mitochondria and clearance of ROS by mitophagy help a cell to maintain mitochondrial homeostasis.

Figure S5. The original uncropped images for immunoblots in the main and supplementary figures.

Fig. 1B

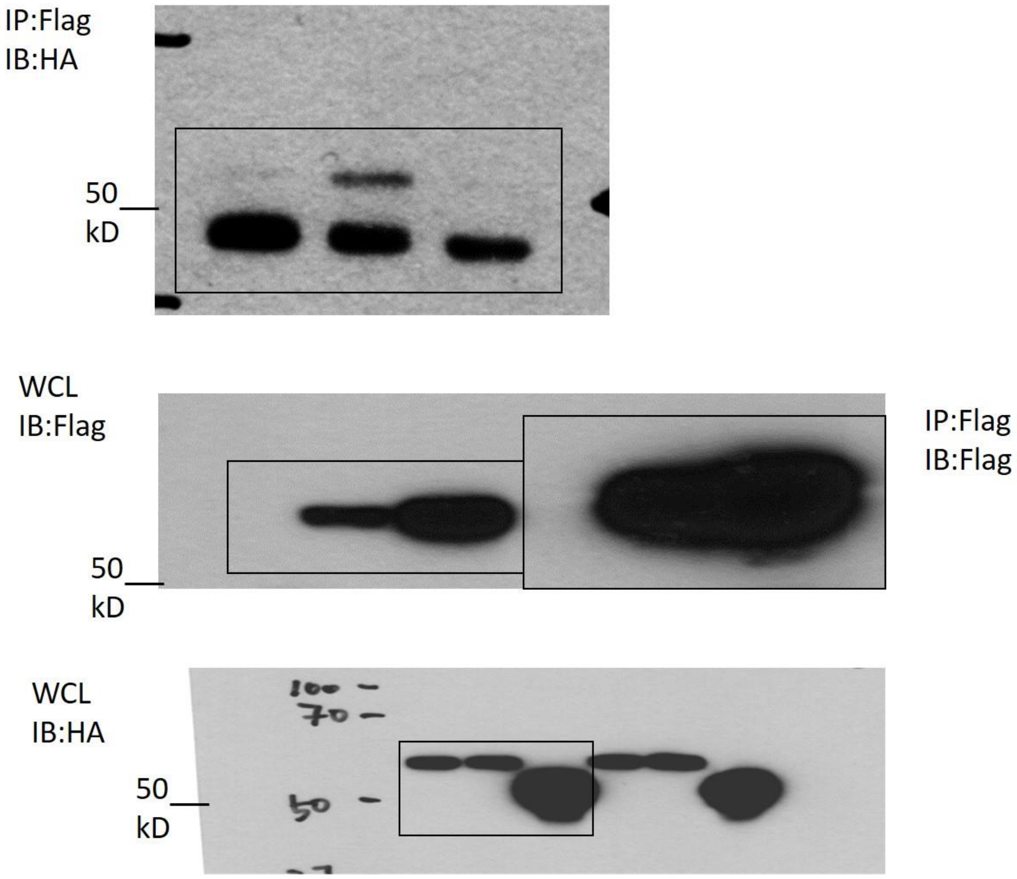

Fig. 1C

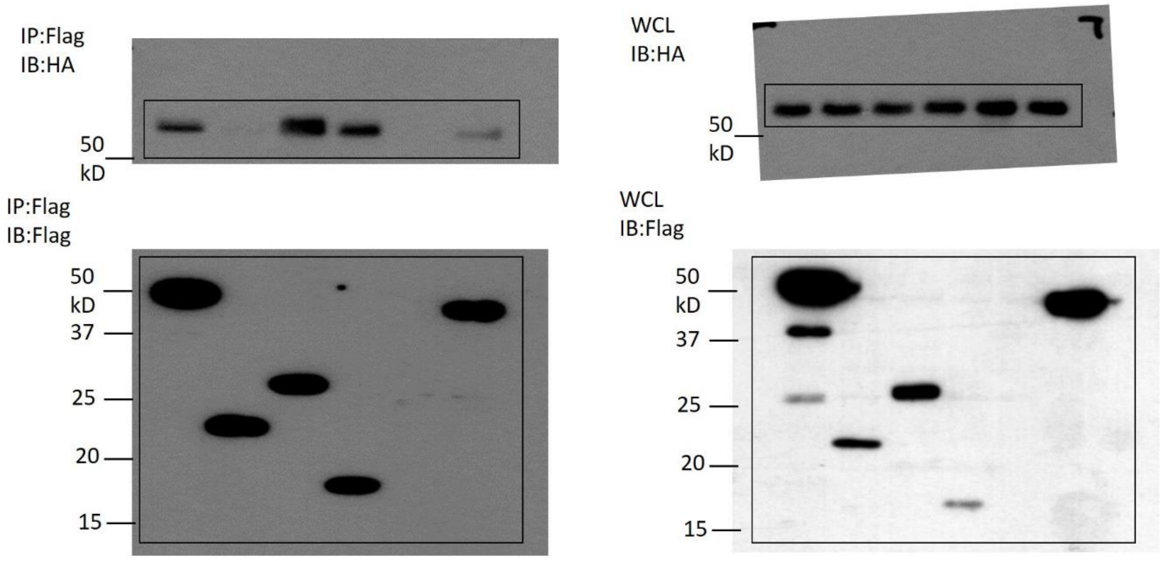

Fig. 2A

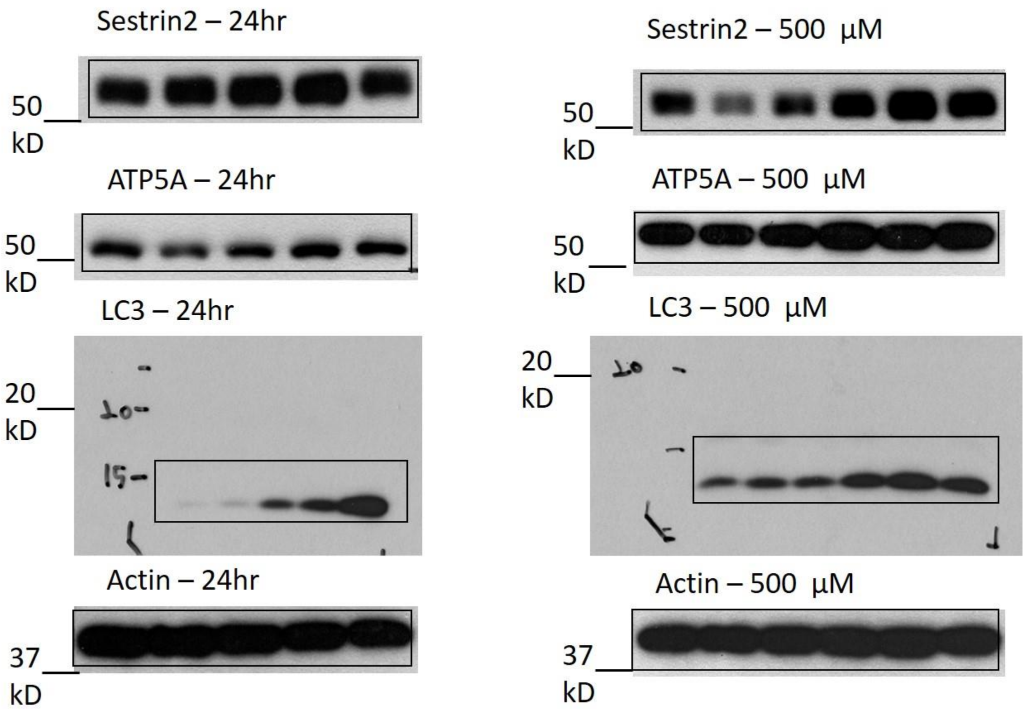
Sestrin2 – 24hr
50 kD
ATP5A – 24hr
50 kD
LC3 – 24hr
20 kD
Actin – 24hr
37 kD
Sestrin2 – 500 μM
50 kD
ATP5A – 500 μM
50 kD
LC3 – 500 μM
20 kD
Actin – 500 μM
37 kD

Fig. 2B

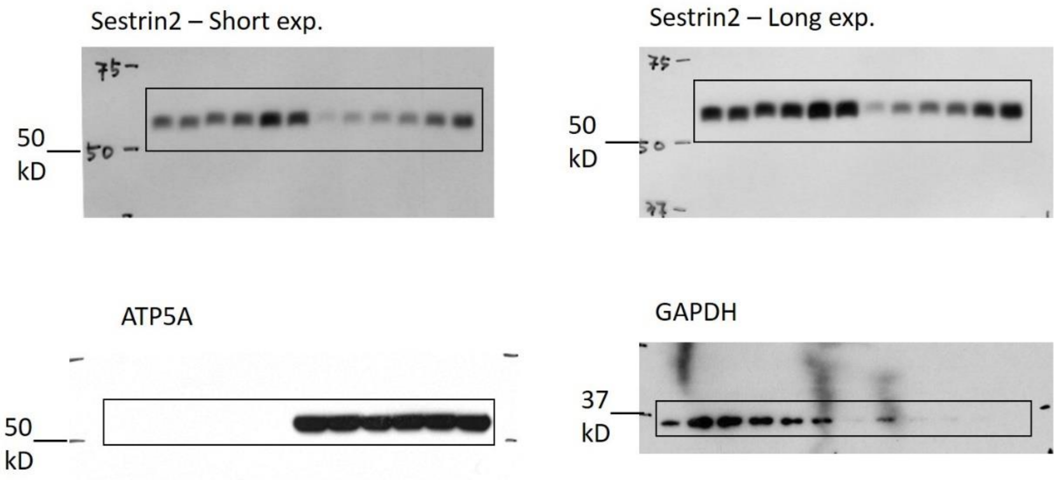
Sestrin2 – Short exp.
50 kD
Sestrin2 – Long exp.
50 kD
ATP5A
50 kD
GAPDH
37 kD

Fig. 2C

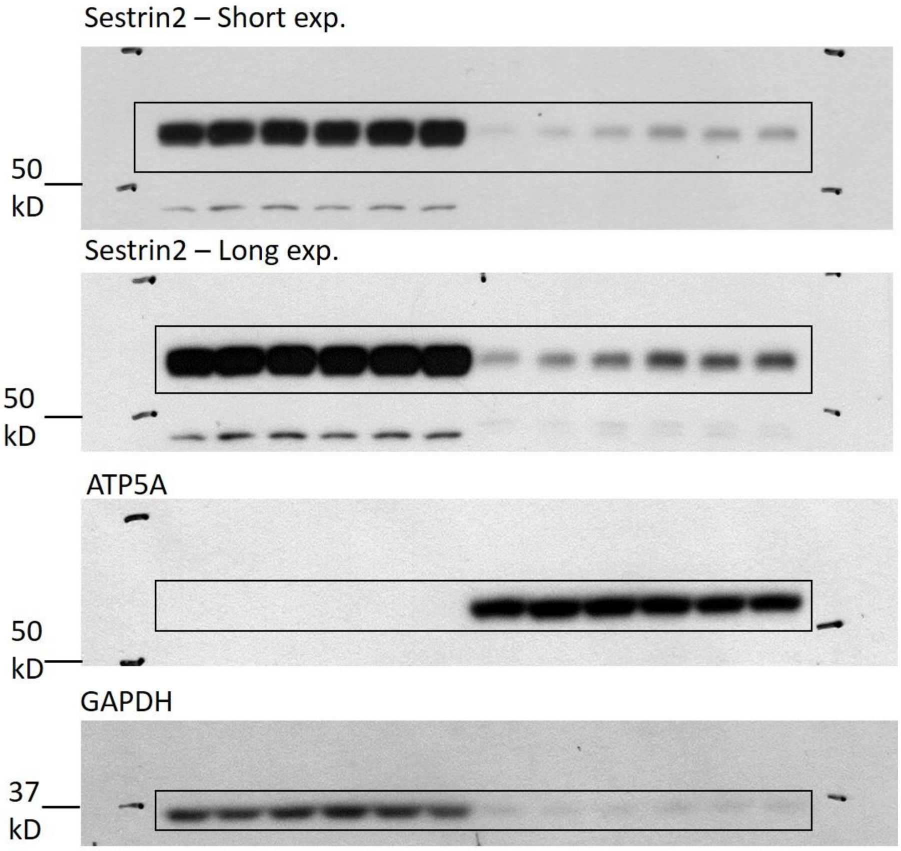
Sestrin2 – Short exp.
50 kD
Sestrin2 – Long exp.
50 kD
ATP5A
50 kD
GAPDH
37 kD

Fig. 2D

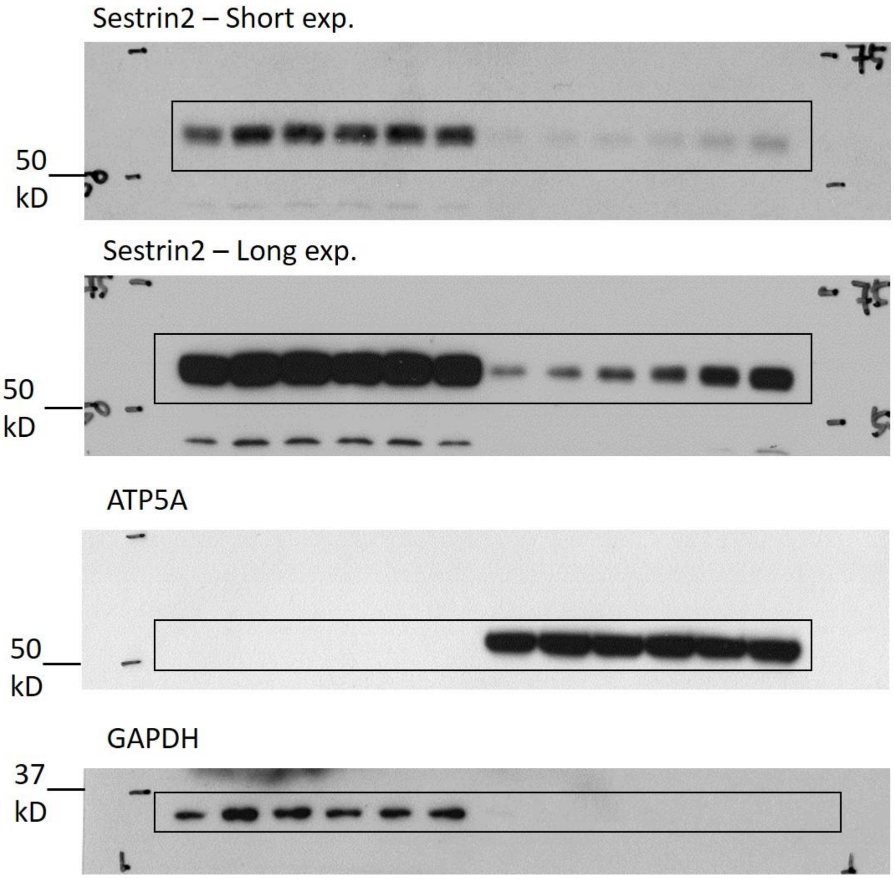
Sestrin2 – Short exp.
50 kD
Sestrin2 – Long exp.
50 kD
ATP5A
50 kD
GAPDH
37 kD

Fig. 2E

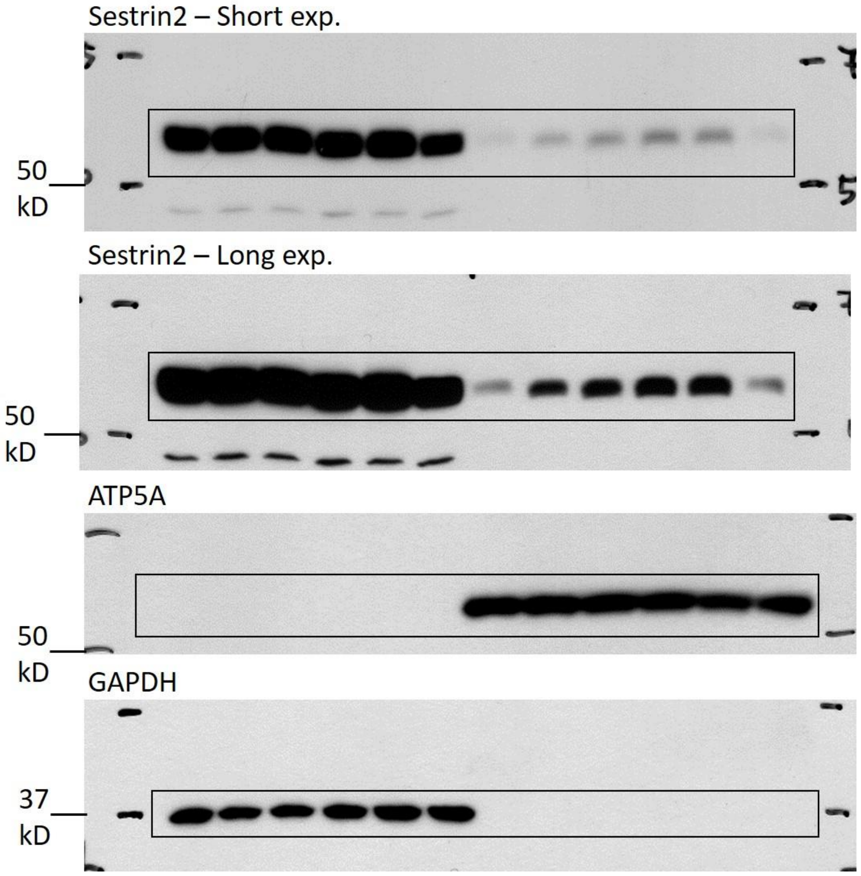
Sestrin2 – Short exp.
50 kD
Sestrin2 – Long exp.
50 kD
ATP5A
50 kD
GAPDH
37 kD

Fig. 2F

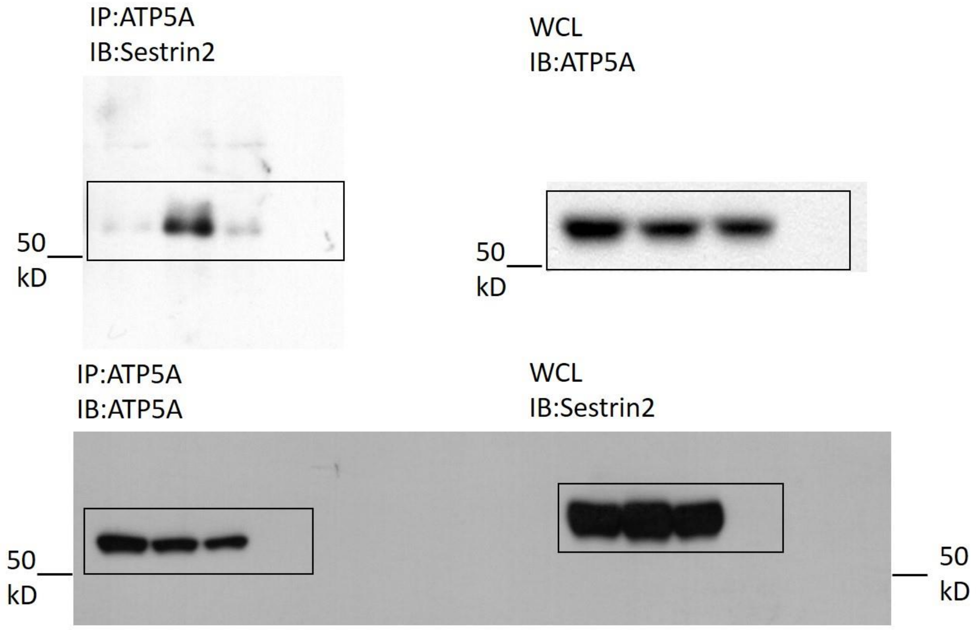
IP:ATP5A
IB:Sestrin2
50 kD
WCL
IB:ATP5A
50 kD
IP:ATP5A
IB:ATP5A
WCL
IB:Sestrin2
50 kD
50 kD

Fig. 3A

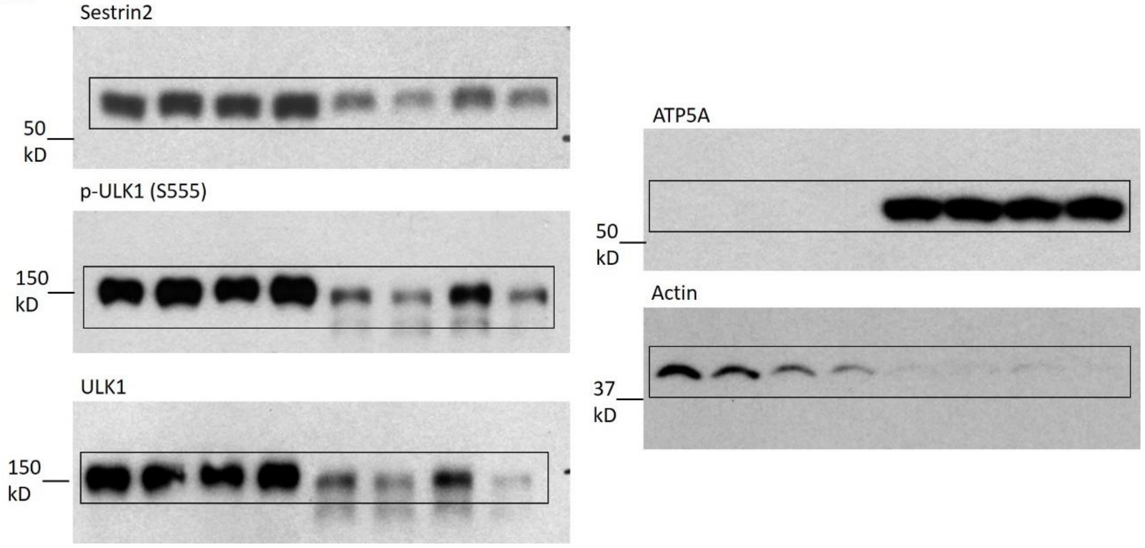
Sestrin2
50
kD
p-ULK1 (S555)
150
kD
ULK1
150
kD
ATP5A
50
kD
Actin
37
kD

Fig. 3B

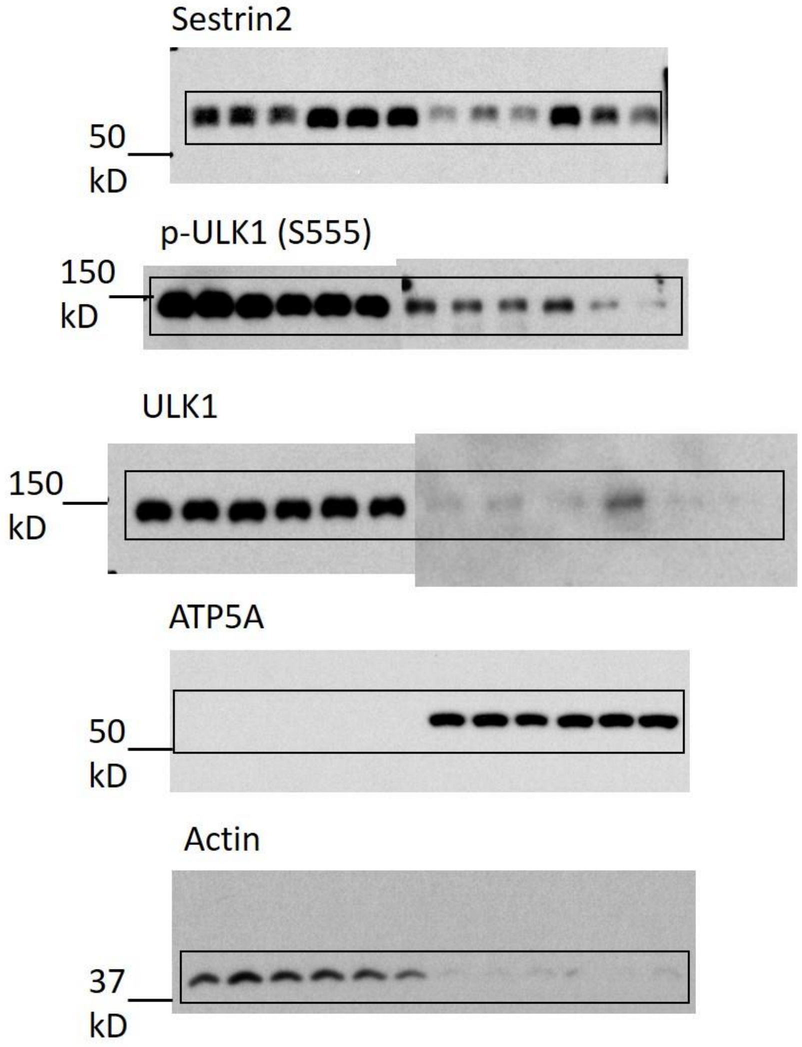
Sestrin2
50
kD
p-ULK1 (S555)
150
kD
ULK1
150
kD
ATP5A
50
kD
Actin
37
kD

Fig. 3C

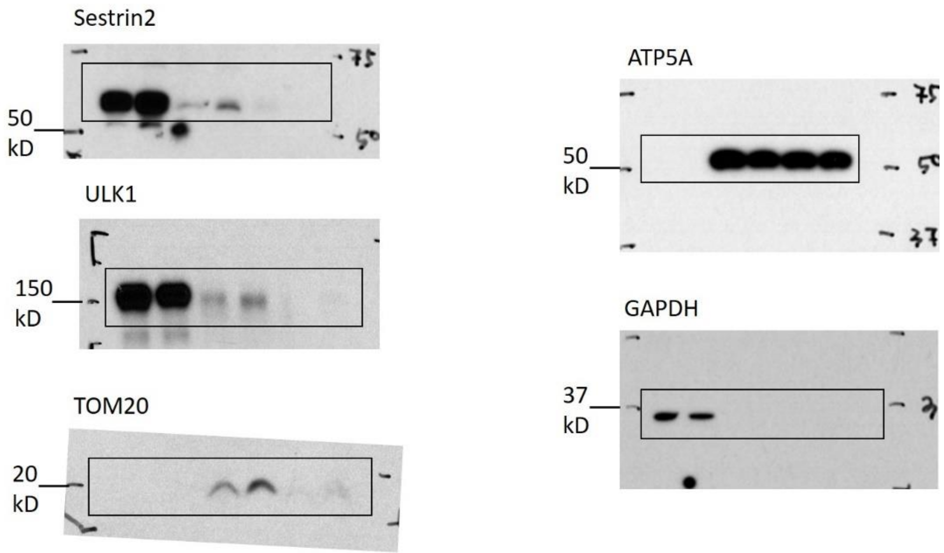
Sestrin2
50 kD
ULK1
150 kD
TOM20
20 kD
ATP5A
50 kD
GAPDH
37 kD

Fig. 3D

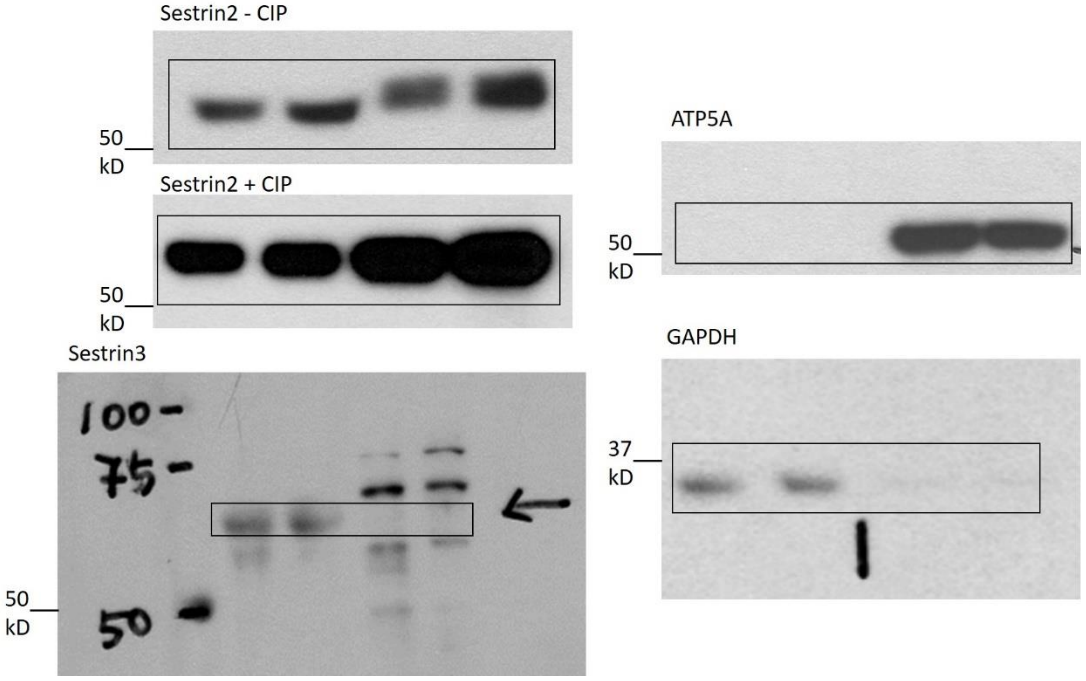
Sestrin2 - CIP
50 kD
Sestrin2 + CIP
50 kD
Sestrin3
100-
75-
50
50 kD
ATP5A
50 kD
GAPDH
37 kD

Fig. 4A

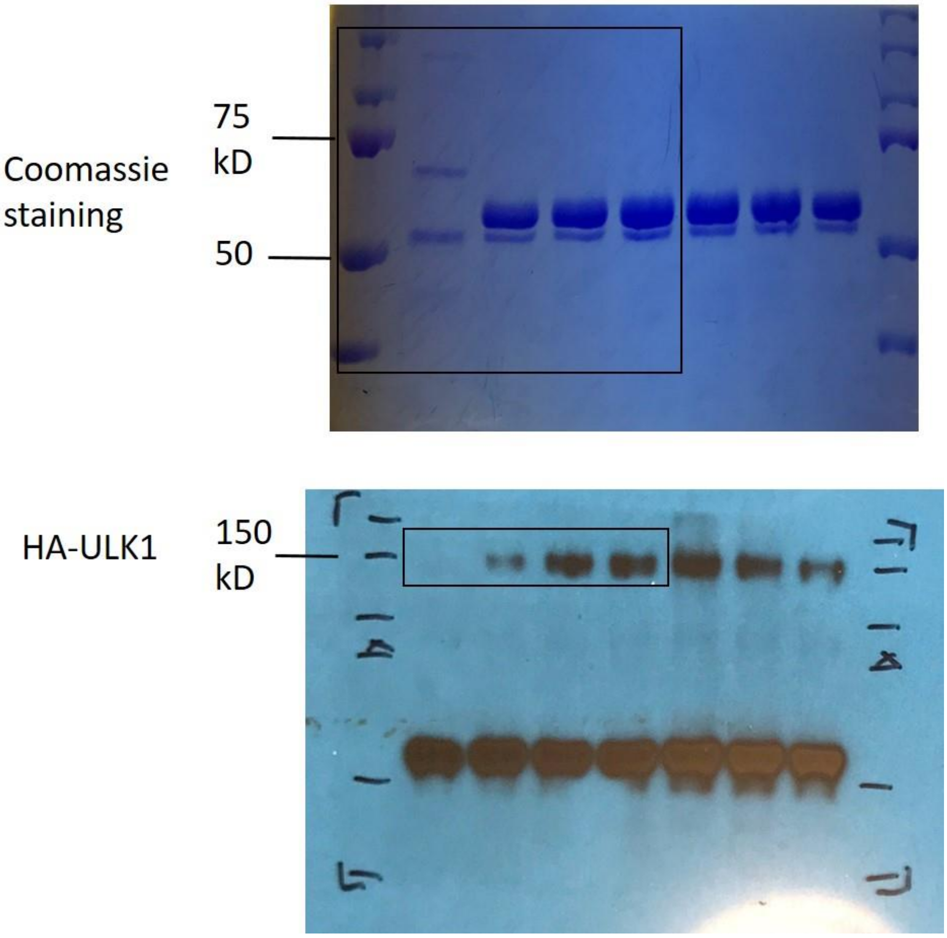

Fig. 6A

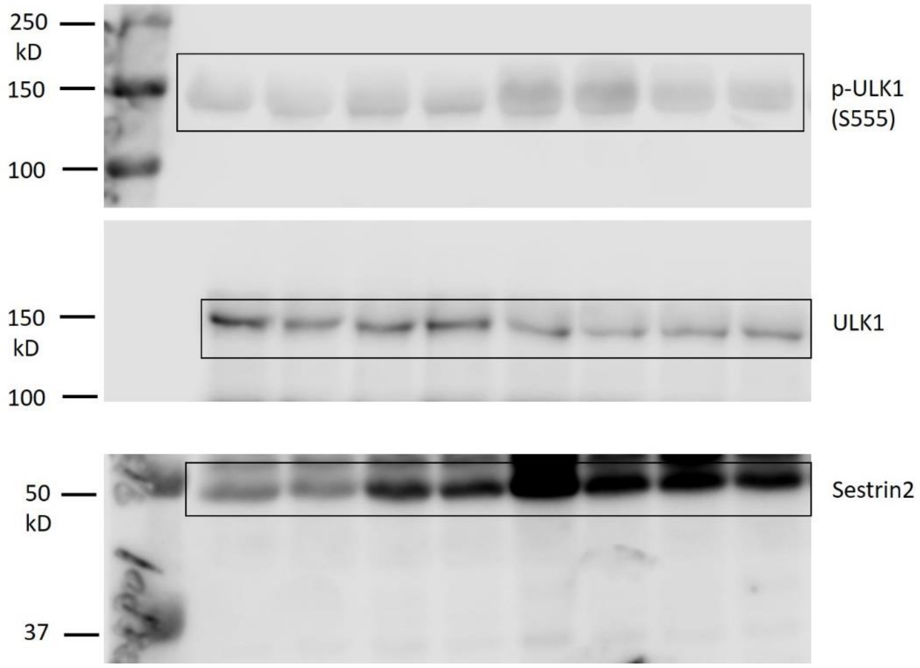

Fig. 6A cont.

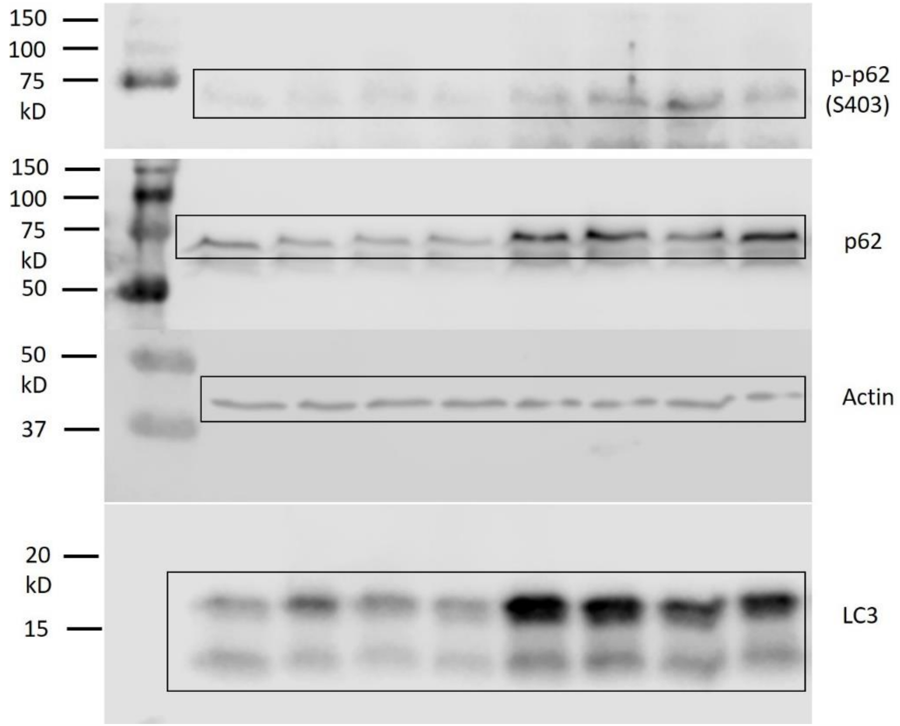

Fig. S1

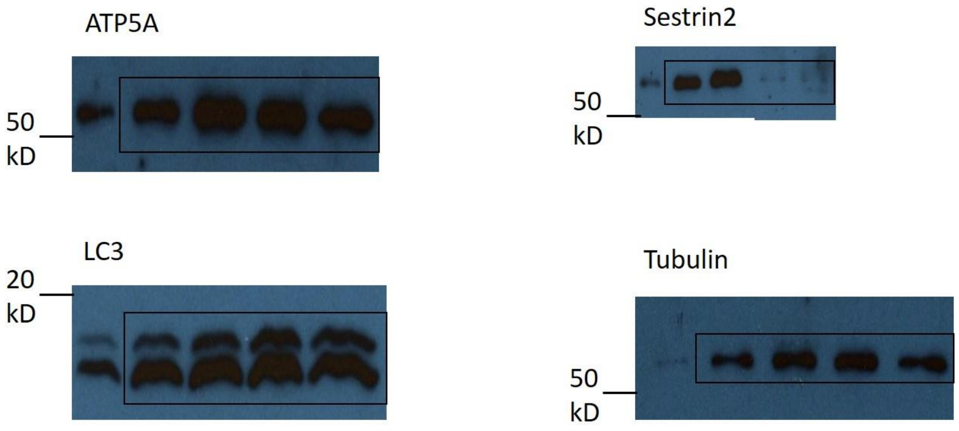

Fig. S2

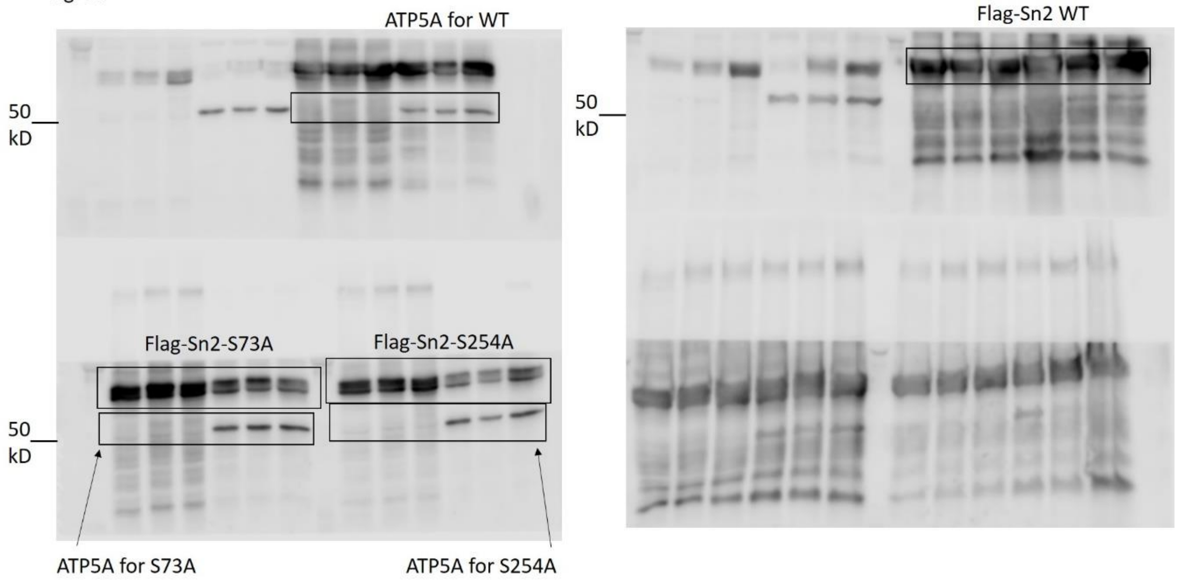

Fig. S2 cont.

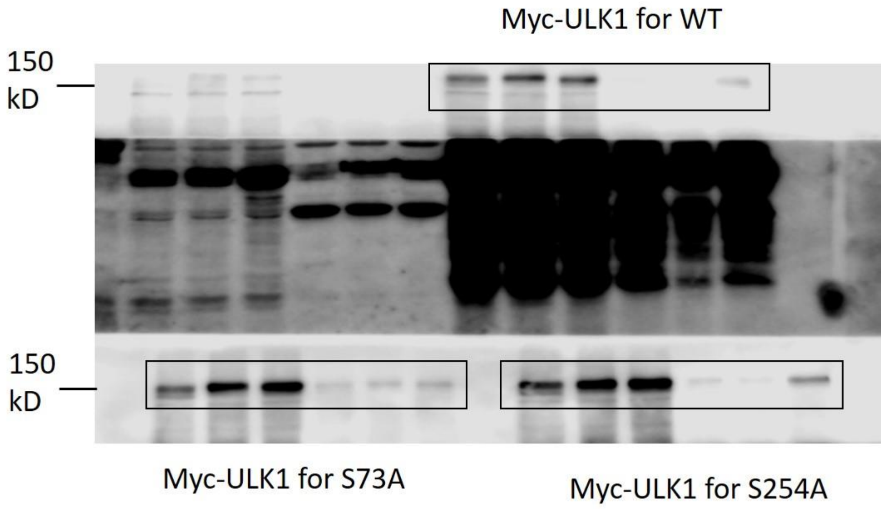

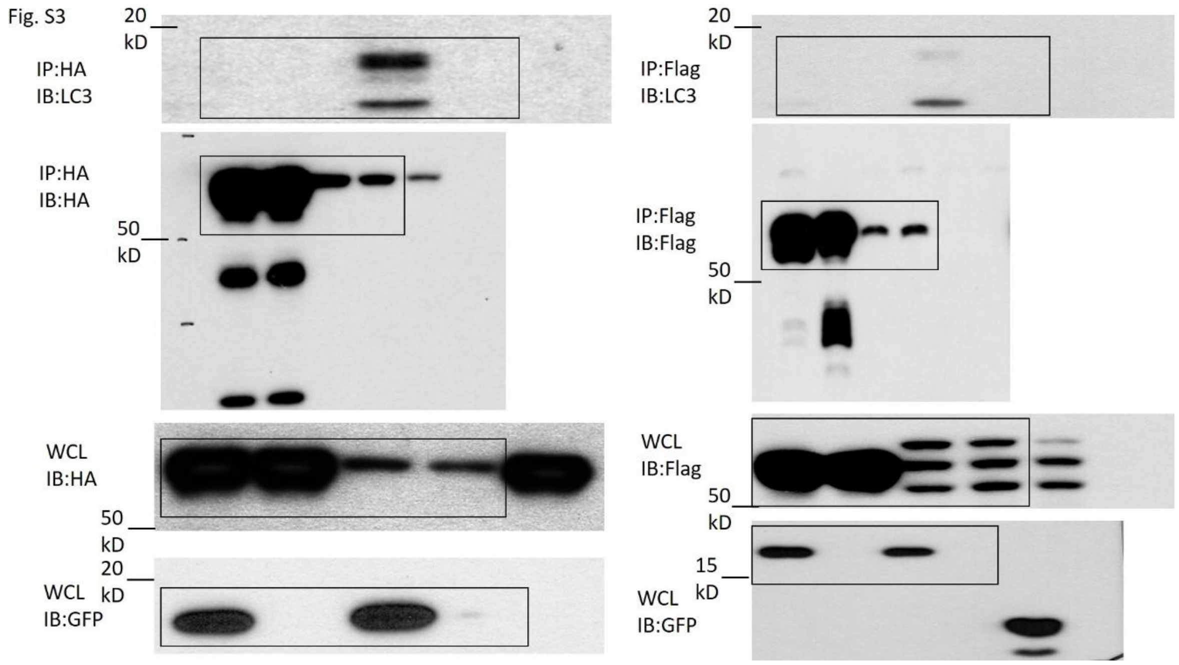
Fig. S3
20 kD
IP:HA
IB:LC3
IP:HA
IB:HA
50 kD
WCL
IB:HA
50 kD
20 kD
WCL
IB:GFP
20 kD
IP:Flag
IB:LC3
IP:Flag
IB:Flag
50 kD
WCL
IB:Flag
50 kD
15 kD
WCL
IB:GFP
